# DDX39B interacts with the pattern recognition receptor pathway to inhibit NF-κB and sensitize to alkylating chemotherapy

**DOI:** 10.1186/s12915-020-0764-z

**Published:** 2020-03-24

**Authors:** Szymon J. Szymura, Giovanna M. Bernal, Longtao Wu, Zhongqin Zhang, Clayton D. Crawley, David J. Voce, Paige-Ashley Campbell, Diana E. Ranoa, Ralph R. Weichselbaum, Bakhtiar Yamini

**Affiliations:** 1 Department of Surgery, Section of Neurosurgery, The University of Chicago, Chicago, IL 60637 USA; 2 Department of Radiation and Cellular Oncology, and The Ludwig Center for Metastasis Research, The University of Chicago, Chicago, IL 60637 USA

**Keywords:** DDX39B, Extracellular matrix, LGP2, NF-κB, PIASx

## Abstract

**Background:**

Nuclear factor-κB (NF-κB) plays a prominent role in promoting inflammation and resistance to DNA damaging therapy. We searched for proteins that modulate the NF-κB response as a prerequisite to identifying novel factors that affect sensitivity to DNA damaging chemotherapy.

**Results:**

Using streptavidin-agarose pull-down, we identified the DExD/H-box RNA helicase, DDX39B, as a factor that differentially interacts with κB DNA probes. Subsequently, using both RNA interference and CRISPR/Cas9 technology, we demonstrated that DDX39B inhibits NF-κB activity by a general mechanism involving inhibition of p65 phosphorylation. Mechanistically, DDX39B mediates this effect by interacting with the pattern recognition receptor (PRR), LGP2, a pathway that required the cellular response to cytoplasmic double-stranded RNA (dsRNA). From a functional standpoint, loss of DDX39B promoted resistance to alkylating chemotherapy in glioblastoma cells. Further examination of DDX39B demonstrated that its protein abundance was regulated by site-specific sumoylation that promoted its poly-ubiquitination and degradation. These post-translational modifications required the presence of the SUMO E3 ligase, PIASx-β. Finally, genome-wide analysis demonstrated that despite the link to the PRR system, DDX39B did not generally inhibit interferon-stimulated gene expression, but rather acted to attenuate expression of factors associated with the extracellular matrix, cellular migration, and angiogenesis.

**Conclusions:**

These results identify DDX39B, a factor with known functions in mRNA splicing and nuclear export, as an RNA-binding protein that blocks a subset of the inflammatory response. While these findings identify a pathway by which DDX39B promotes sensitization to DNA damaging therapy, the data also reveal a mechanism by which this helicase may act to mitigate autoimmune disease.

## Background

Nuclear factor-κB (NF-κB) is an inducible transcription factor that plays a prominent role in the cellular response to stress [1]. In the unstimulated state, latent NF-κB subunits are generally maintained in the cytoplasm in association with inhibitor-κB (IκB) proteins. While phosphorylation-dependent IκB degradation is a central node in the regulation of NF-κB, direct p65 (RELA) phosphorylation also activates NF-κB [2]. Among the many inducing stimuli, factors such as cytosolic nucleic acid activate NF-κB as part of the innate immune system [3]. While this response is essential for combating exogenous pathogens, endogenously produced nucleic acids can also activate NF-κB signaling. Such endogenous activation can lead to chronic inflammation that promotes autoimmune disease, cancer, and resistance to therapy [4, 5]. Given the harmful effects of un-controlled NF-κB activity and inflammation, a variety of mechanisms have evolved to prevent excess activation of NF-κB by endogenous sources [6]. In this regard, recent studies have highlighted the role of RNA-binding proteins (RBPs) in preventing inflammation due to endogenously produced RNA [7, 8].

DExD/H-box RNA helicases are a family of proteins that comprise a large proportion of the receptors that sense cytoplasmic double-stranded RNA (dsRNA) and DNA [9, 10]. DDX39B (Bat1, UAP56) is an evolutionarily conserved DECD-box helicase that regulates multiple aspects of RNA metabolism. While this protein was initially identified as a pre-mRNA splicing factor [11], subsequent studies demonstrated that it was also involved in mRNA nuclear export [12, 13]. Despite the above, depletion of DDX39B in mammalian cells does not generally block protein synthesis [14], possibly because of the presence of a similar protein, DDX39A (URH49) [15]. In addition to mRNA processing, DDX39B was independently identified as Bat1 (HLA-B-associated transcript 1), a helicase whose gene is located in the central MHC region [16, 17], a locus associated with a variety of autoimmune and inflammatory diseases [18–21]. Studies with Bat1 demonstrated that it downregulated production of pro-inflammatory cytokines [22] and polymorphisms of *DDX39B/BAT1* linked with reduced activity have also been associated with autoimmune disease [23, 24].

NF-κB plays a complex role in the response to DNA damage and alkylating chemotherapy [25–27]. Here, we set out to identify novel factors that modulate NF-κB signaling and identified DDX39B as a factor that differentially bound κB DNA probes. DDX39B decreased NF-κB activity and promoted sensitivity to alkylating chemotherapy in GBM cells. Mechanistically, DDX39B inhibited NF-κB via interaction with the pattern recognition receptor (PRR), laboratory of genetics and physiology 2 (LGP2, DHX58). Ultimately, genome-wide analysis demonstrated that loss of DDX39B induced the expression of factors that regulate the extracellular matrix (ECM) and promote angiogenesis. These studies indicate that DDX39B attenuates the response to endogenous dsRNA, and suggest that loss of DDX39B and activation of this inflammatory pathway is detrimental to the efficacy of DNA damaging therapy.

## Results

### DDX39B inhibits NF-κB activity

**Fig. 1:**
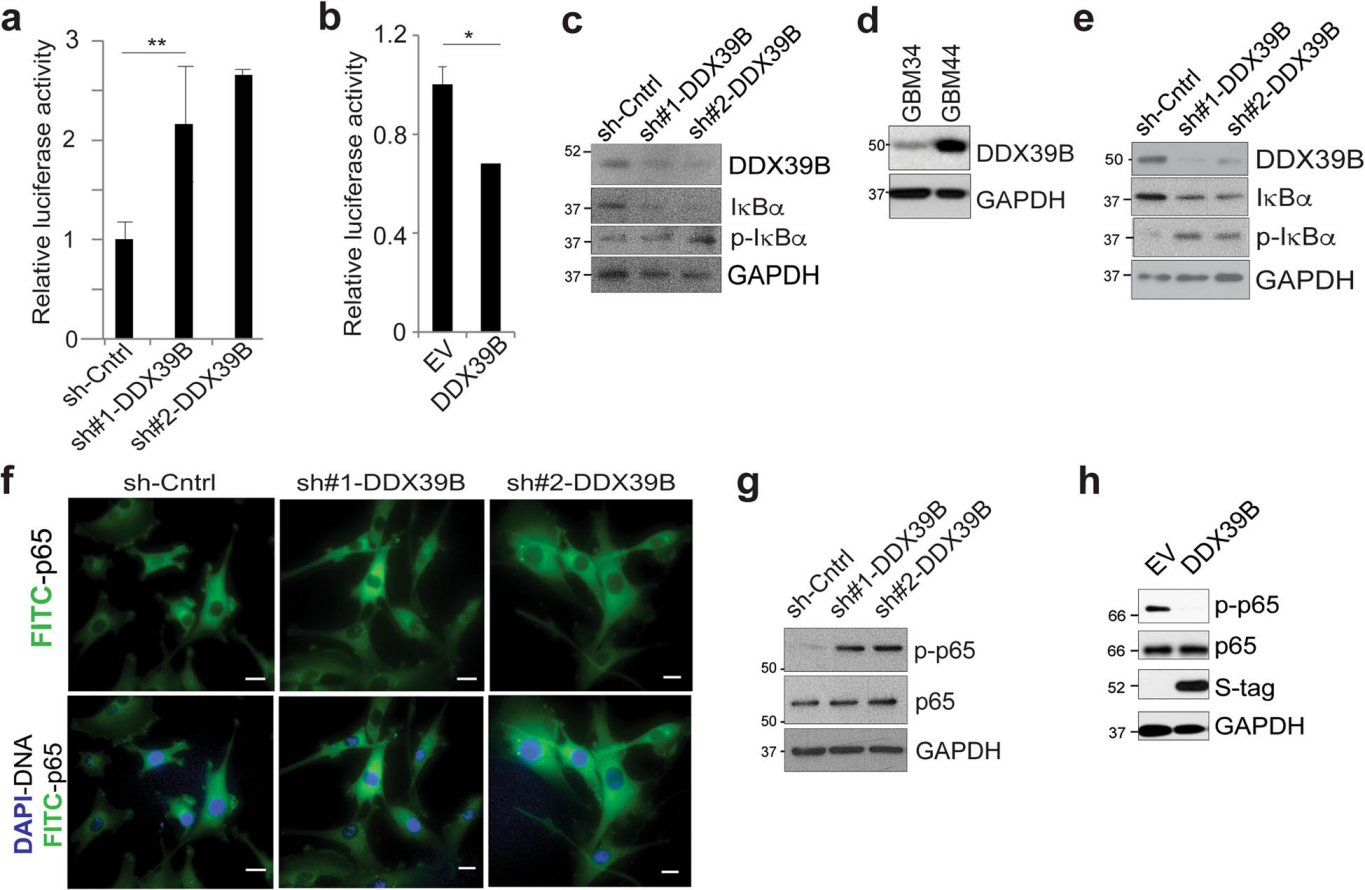
DDX39B inhibits NF-κB activity. **a** Luciferase assay using a reporter bearing -1C κB-sites in A172 cells expressing two independent sh-DDX39B constructs or a control vector. Data show mean value normalized to EV, ± SEM from two independent experiments. **b** Luciferase assay in U87 cells transfected with empty vector (EV) or DDX39B. Data show mean value normalized to EV, ± SEM from two independent experiments. **c** Immunoblot (IB) in A172 GBM cells expressing two sh-DDX39B constructs or sh-control. IB was performed with anti-phospho-IκBα, anti-IκBα, or anti-GAPDH as loading control. **d** IB in GBM34 and GBM44 GSCs probed with anti-DDX39B. **e** Immunoblot in GBM44 GSCs expressing shRNA constructs as in **c**. IB performed as in **c**. **f** Representative immunofluorescence (IF) analysis of endogenous p65 in A172 cells expressing sh-DDX39B. Nuclei were counterstained with 4′,6-diamidino-2-phenylindole (DAPI). Scale bar, 10 μm. **g** IB in GBM44 GSCs from **e** expressing shRNA constructs probed with anti-phospho-p65 and anti-p65 antibodies. **h** IB in GBM34 GSCs stably expressing empty vector (EV) or S-tagged DDX39B with anti-phospho-p65 and anti-p65. *P < 0.05, **P < 0.01 (two-tailed *t* test)

Given the importance of the κB-site in regulating the response to alkylating chemotherapy [28], we performed streptavidin-agarose pull-down using biotin-tagged oligonucleotides containing κB binding sequences. Gel electrophoresis and silver staining of the pull-down product revealed a band that differentially bound κB DNA probes that vary only at the − 1 nucleotide (Additional file 1: Fig. S1a). MS/MS analysis of this band identified DDX39B as one of the only non-keratin peptides (Additional file 1: Fig. S1b). To validate DNA binding of DDX39B, we expressed and purified DDX39B protein (Additional file 1: Fig. S1c) and found that this protein bound to the -1C probe more than the -1A probe (Additional file 1: Fig. S1d). Given the propensity of DDX39B to bind κB DNA, we examined whether loss of DDX39B altered NF-κB activity. Several regions of DDX39B were targeted with short-hairpin (sh) vectors and a series of cell lines expressing either control or sh-DDX39B constructed (Additional file 1: Fig. S1e). Using a luciferase reporter under the control of the -1C κB-site, we found that loss of DDX39B increased NF-κB activity compared to control (Fig. 1a). Conversely, overexpression of DDX39B reduced NF-κB activity from this reporter (Fig. 1b). Of note, DDX39B also inhibited expression from a reporter under the control of a -1A κB-site (Additional file 1: Fig. S1f).

To examine the mechanism for inhibition of NF-κB by DDX39B, we analyzed IκBα, a primary regulator of the NF-κB response. Knockdown of *DDX39B* in GBM cells resulted in a decrease in IκBα protein (Fig. 1c). This decrease was not due to reduced mRNA expression as loss of *DDX39B* actually increased *NFKBIA* mRNA (Additional file 1: Fig. S2a). In addition, loss of *DDX39B* did not alter the fraction of *NFKBIA* in the cytoplasm (Additional file 1: Fig. S2b), an important finding given that DDX39B regulates mRNA nuclear export [13]. Given these findings, we examined IκBα phosphorylation and noted that loss of *DDX39B* resulted in increased phosphorylation (Fig. 1c). To confirm this unexpected finding in distinct cells, we used the patient-derived glioma stem-like cells (GSCs), GBM34 and GBM44 [27, 29]. We first determined the basal DDX39B protein abundance in these cells and noted that GBM44 GSCs have substantially more DDX39B than GBM34 (Fig. 1d). We then knocked down *DDX39B* in GBM44 GSCs and again observed both increased IκBα phosphorylation and decreased steady state IκBα protein (Fig. 1e). These results suggested that the decrease in IκBα protein was related to its phosphorylation. These findings raised the question of whether the increase in NF-κB activity with loss of DDX39B was due to increased nuclear p65 and p50 protein. Both immunoblot and immunofluorescence (IF) analysis did not show an observable change in nuclear p65 or p50 with loss of *DDX39B* (Fig. 1f and Additional file 1: Fig. S2c), suggesting that additional alterations mediated the change in NF-κB activity. Given the increase in IκBα phosphorylation, we examined p65 phosphorylation. Knockdown of *DDX39B* resulted in a substantial increase in p65 Ser536 phosphorylation in both GBM44 GSCs (Fig. 1g) and A172 GBM cells (Additional file 1: Fig. S2d). Conversely, overexpression of DDX39B in GBM34 GSCs that have low baseline DDX39B attenuated p65 phosphorylation (Fig. 1h). These findings indicate that loss of DDX39B increases NF-κB activity via a general effect on p65 phosphorylation.

### DDX39B blocks NF-κB as part of the response to RNA

**Fig. 2:**
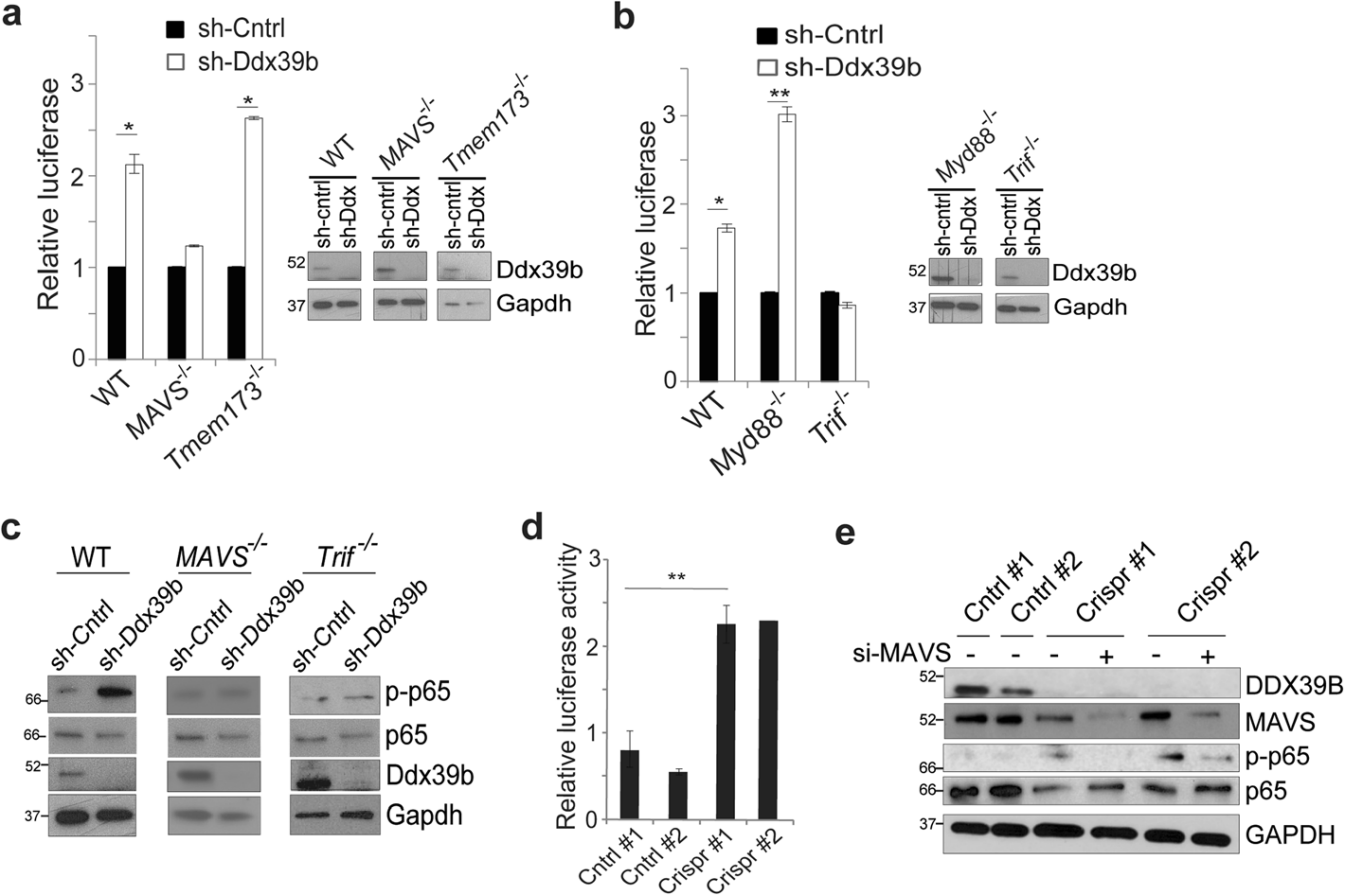
DDX39B regulates NF-κB via MAVS and TRIF. **a** Luciferase assay using the -1C reporter in primary mouse embryonic fibroblasts (MEFs) isolated from wild-type, *MAVS*^−/−^, and *Tmem173*^−/−^ mice following infection with sh-Ddx39b (Ddx) or non-targeting control. Data show mean relative value, ± SEM of two independent experiments. Inset: IB with anti-DDX39B antibody. **b** Luciferase assay performed as in **a** using wild-type, *Myd88*^−/−^, and *Trif*^−/−^ MEFs. Data show mean relative value, ± SEM of two independent experiments. **c** IB in the indicated MEFs infected with sh-Ddx39b or non-targeting control using anti-phospho-p65 antibody. **d** Luciferase assay in U87 control or *DDX39B* CRISPR cells. Data show mean relative value, ± SEM of two independent experiments. **e** IB in U87 control and *DDX39B* CRISPR clones transfected with si-MAVS or si-control probed with the indicated antibodies. *P < 0.05, **P < 0.01 (two-tailed *t* test)

The increase in p65 phosphorylation in the absence of external stimulation suggested that DDX39B modulated NF-κB via an internal signaling response. Consistent with such a hypothesis, DDX39B was recently linked to the PRR pathway [8]. Given that DDX39B is known to bind mRNA and we find that it also binds DNA (Additional file 1: Fig. S1d), we examined whether the response to either of these nucleic acids mediated the effect of DDX39B on NF-κB. To this end, primary mouse embryonic fibroblasts (MEFs) deleted of either stimulator of IFN genes (STING), encoded by *Tmem173*, or mitochondrial antiviral signaling protein (MAVS) were obtained. These adaptor proteins mediate the response to cytoplasmic DNA and dsRNA, respectively. In wild-type (wt) MEFs, like human cells, knockdown of *Ddx39b* increased NF-κB activity (Fig. 2a). However, whereas loss of *Ddx39b* induced NF-κB in *Tmem173*^*−/−*^ MEFs, in *MAVS*^*−/−*^ MEFs, depletion of *Ddx39b* did not (Fig. 2a). These findings indicated that DDX39B modulated the NF-κB response to cytoplasmic dsRNA, not DNA. In addition to MAVS, two other adapter proteins mediate signaling associated with cytosolic RNA, myeloid differentiation primary response 88 (MYD88) and TIR-domain-containing adapter-inducing interferon-β (TRIF) [30]. While knockdown of *Ddx39b* increased NF-κB activity in *Myd88*^*−/−*^ MEFs, in *Trif*^*−/−*^ MEFs, no increase in NF-κB activity was seen (Fig. 2b). To further study these factors, we examined changes in p65 phosphorylation. While p65 phosphorylation was increased in wt MEFs following knockdown of *Ddx39b*, in both *MAVS*^*−/−*^ and *Trif*^*−/−*^ MEFs, no increase in phospho-p65 was seen (Fig. 2c). These findings support the role of MAVS and TRIF in mediating the regulation of NF-κB by DDX39B.

As a further specificity control to ensure that the changes in NF-κB were not a consequence of lentiviral infection, especially given that PRR signaling is modulated by viral infection, we used CRISPR/Cas9 technology to delete DDX39B in U87 GBM cells. Several CRISPR clones were isolated, and loss of DDX39B verified by immunoblot (Additional file 1: Fig. S2e). Similar to shRNA knockdown, CRISPR-mediated depletion of DDX39B resulted in both increased NF-κB activity and increased p65 phosphorylation (Fig. 2d and Additional file 1: Fig. S2f) without any appreciable change in p65 nuclear translocation (Additional file 1: Fig. S2g). Using these clones, we examined the role of MAVS in human cells and found that while depletion of *DDX39B* by CRISPR increased p65 phosphorylation compared to control, knockdown of *MAVS*, in two independent clones, reduced this phosphorylation (Fig. 2e). Consistent with this finding, knockdown of MAVS also blocked the increase in NF-κB activity induced by loss of DDX39B (Additional file 1: Fig. S2h). In addition, loss of DDX39B by CRISPR rendered U87 cells highly resistant to TMZ, an effect that was reversed by re-expression of DDX39B (Additional file 1: Fig. S2i). These results indicate that DDX39B blocks NF-κB activity by a mechanism involving the response to dsRNA.

### DDX39B inhibits NF-κB in association with LGP2

**Fig. 3:**
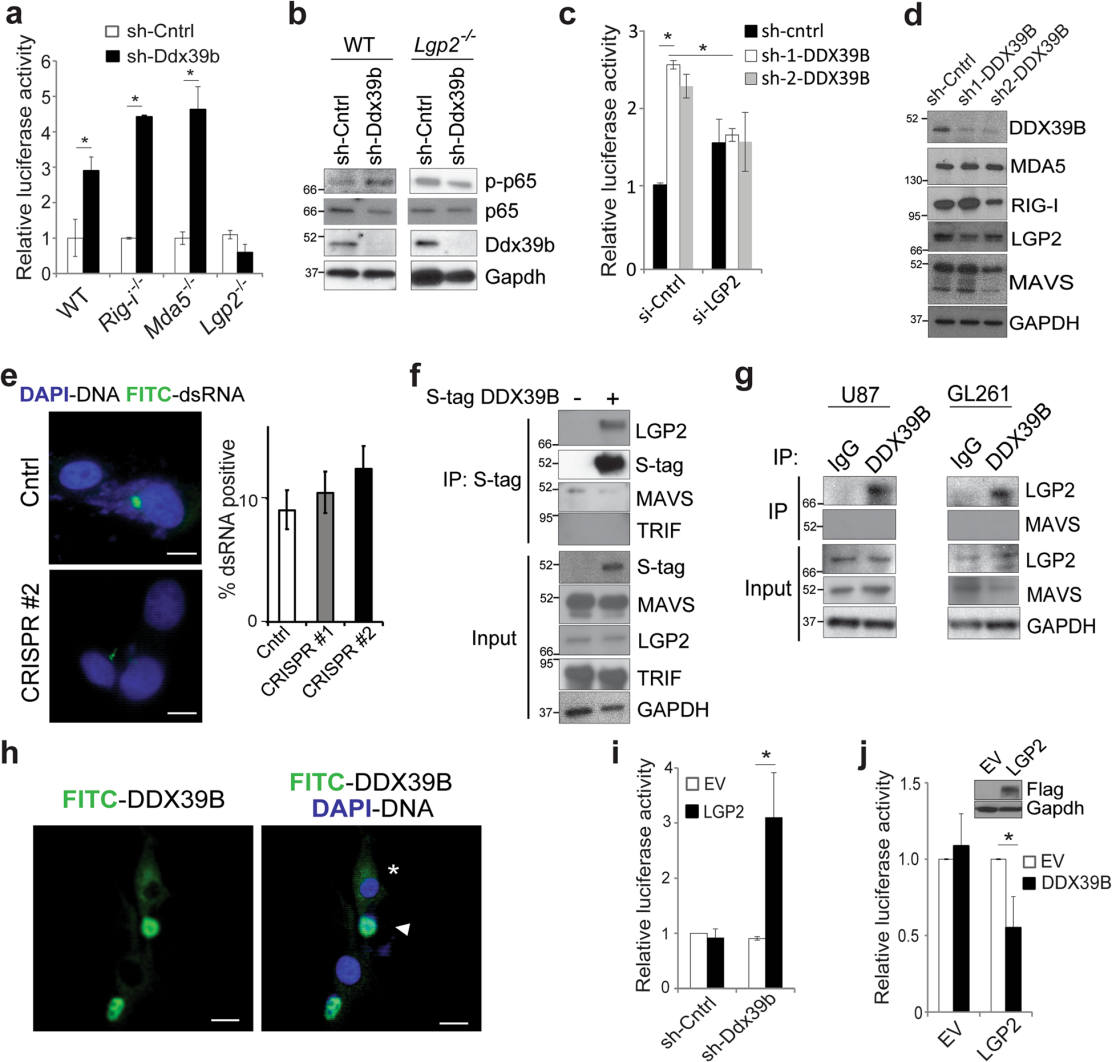
DDX39B modulates NF-κB activity in association with LGP2. **a** Luciferase assay using the -1C reporter in wild-type, *Rig-I*^−/−^, *Mda5*^−/−^, and *Lgp2*^−/−^ MEFs following infection with sh-Ddx39b or a non-targeting control. Data show mean relative value, ± SEM of two independent experiments. **b** Immunoblot (IB) in the indicated MEFs infected with sh-Ddx39b or non-targeting control using anti-phospho-p65 antibody. **c** Luciferase assay in U87 cells stably expressing sh-DDX39B or non-targeting control transfected with si-LGP2 or si-control. Data show mean value, ± SEM of two independent experiments. **d** IB in A172 cells expressing sh-DDX39B or non-targeting control probed with the indicated antibodies. **e** Immunofluorescence (IF) staining (left) and quantification (right) of dsRNA in U87 DDX39B CRISPR cells. Data show mean value, ± SD of ten view fields, repeated. Scale bar, 10 μm. **f** Co-immunoprecipitation (Co-IP) in U87 cells transfected with empty vector or S-tagged DDX39B. IP with S-protein agarose followed by IB using the indicated antibodies. Input probed as shown. **g** Co-IP in U87 cells (left) and GL261 mouse GBM cells (right) following IP with anti-DDX39B or control IgG and IB with the indicated antibodies. Input probed as shown. **h** IF staining for endogenous DDX39B (FITC) in U87 cells. DAPI counter stain. Scale bar, 10 μm. Asterisk, cytoplasmic DDX39B; arrowhead, nuclear DDX39B. **i** Luciferase assay in *Lgp2*^−/−^ MEFs infected with sh-DDX39B shRNA or sh-control and transfected with empty vector (EV) or LGP2. Data show mean value, ± SEM of three independent experiments. **j** Luciferase assay in *Lgp2*^−/−^ MEFs cells infected with EV or S-tagged DDX39B and transfected with an independent EV or FLAG-LGP2. Data show mean value, ± SEM of three independent experiments. Inset: IB with anti-FLAG. *P < 0.05 (two-tailed *t* test)

MAVS primarily mediates signaling downstream of the dsRNA sensors, retinoic acid-inducible gene I (RIG-I), melanoma differentiation-associated gene 5 (MDA5), and LGP2 [30]. We examined whether these RIG-I-like receptors (RLRs) were required for the effect of DDX39B on NF-κB. Although knockout of either *Rig-i* or *Mda5* in MEFs did not attenuate the increase in NF-κB induced by sh-Ddx39b, deletion of *Lgp2* did (Fig. 3a), suggesting that DDX39B acted via LGP2 to regulate NF-κB activity. Consistent with this, in *Lgp2*^*−/−*^ MEFs, knockdown of *Ddx39b* failed to increase p65 phosphorylation (Fig. 3b). To examine whether LGP2 was required for the effect of DDX39B in human cells, we used siRNA targeting human *LGP2*. Whereas sh-DDX39B increased NF-κB activity in cells expressing a control siRNA, in the presence of si-LGP2, knockdown of *DDX39B* did not increase NF-κB activity (Fig. 3c). These results indicate that LGP2, but not other RLRs, is required for the increase in NF-κB activity induced by DDX39B loss.

We next examined whether loss of DDX39B altered the abundance of the specific proteins involved in this pathway. Knockdown of *DDX39B* did not significantly change the protein or mRNA level of LGP2, MAVS, RIG-I, or MDA5 (Fig. 3d and Additional file 1: Fig. S3a). Protein kinase R (PKR) is another PRR previously shown to be activated by loss of DDX39B [31]. We examined whether PKR was required for the effect of DDX39B and found that knockdown of *PKR* did not significantly alter activation of NF-κB by *DDX39B* loss (Additional file 1: Fig. S3b). Similarly, toll-like receptor 3 (TLR3) was not required for this response (Additional file 1: Fig. S3b), an important observation given that TRIF mediates signaling downstream of TLR3 in response to endosomal dsRNA [30]. In addition, given the role of LGP2 and MAVS in mediating the response to dsRNA, we examined whether the increase in NF-κB was due to a change in the amount of cytoplasmic dsRNA, a finding previously reported with knockdown of *DDX39B* in the setting of influenza A infection [31]. Using a monoclonal antibody against dsRNA, we found that loss of *DDX39B* did not significantly increase the amount of cytoplasmic dsRNA (Fig. 3e and Additional file 1: Fig. S3c). Together, these findings indicate that although DDX39B regulated NF-κB via the PRR response to dsRNA, this was not a non-specific effect related to changes in dsRNA signaling.

We next examined whether DDX39B interacted with the PRRs involved. Overexpressed DDX39B did not associate with either MAVS or TRIF but did interact with LGP2 (Fig. 3f). Moreover, this interaction was evident even in the presence of nuclease, ruling out a nucleic acid bridge as the mechanism (Additional file 1: Fig. S3d). Subsequently, we confirmed the endogenous association of DDX39B with LGP2 in both human and mouse cells (Fig. 3g). Importantly, although LGP2 is cytoplasmic and DDX39B nuclear, we found that endogenous DDX39B was also present in the cytoplasm (Fig. 3h), a finding previously noted [32–34]. Finally, to validate the role of LGP2 in promoting NF-κB activation in the setting of *DDX39B* loss, we re-expressed LGP2 in *Lgp2*^*−/−*^ MEFs. Whereas knockdown of *DDX39B* did not increase NF-κB in *Lgp2*^*−/−*^ MEFs, when LGP2 was re-expressed, loss of *DDX39B* induced an increase in NF-κB (Fig. 3i). In addition, while overexpression of DDX39B in *Lgp2*^*−/−*^ MEFs did not inhibit NF-κB activity, re-expression of LGP2 in these cells enabled inhibition of NF-κB by DDX39B expression (Fig. 3j). In sum, these results demonstrate that DDX39B inhibits NF-κB by a mechanism involving its interaction with LGP2.

### DDX39B promotes cytotoxicity by temozolomide in GBM cells

**Fig. 4:**
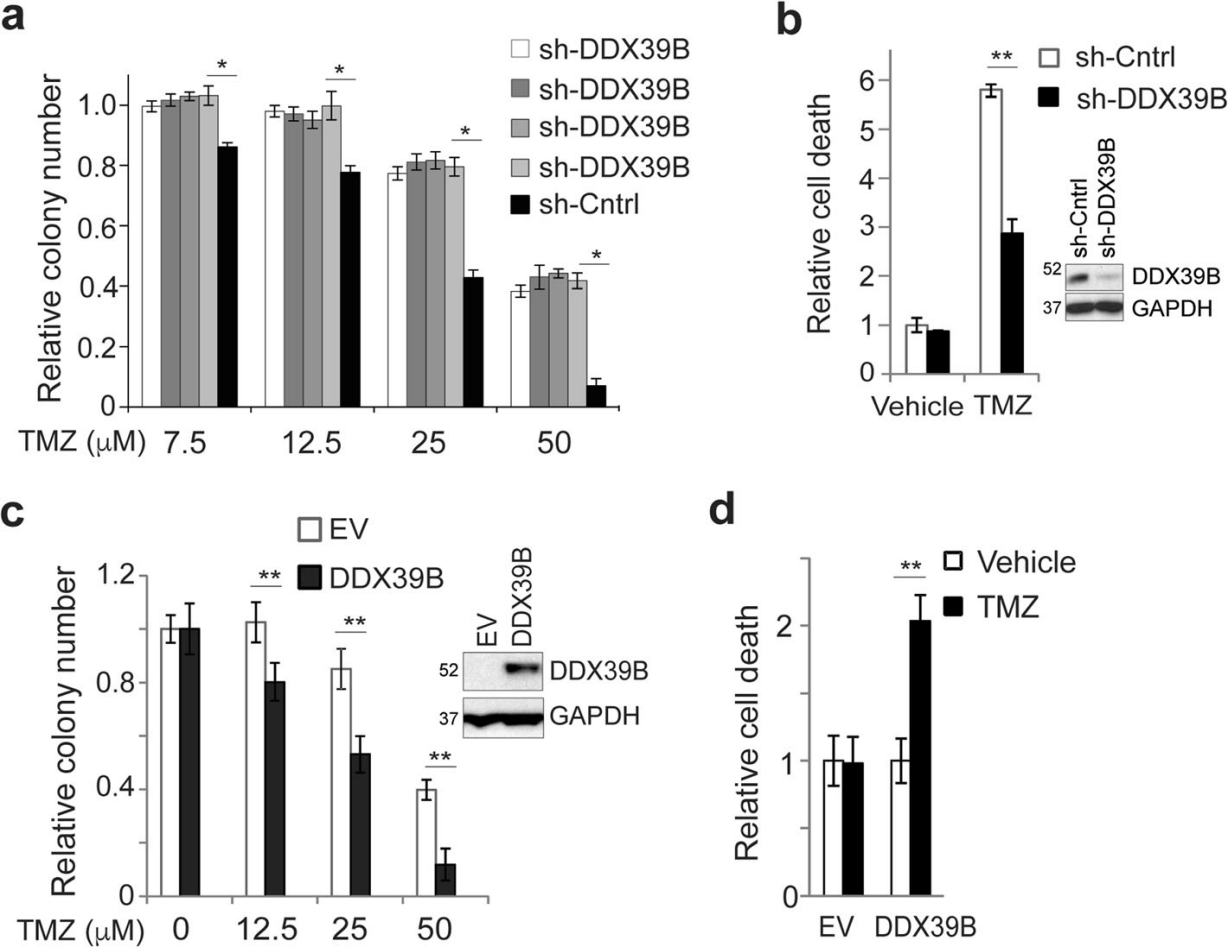
DDX39B modulates resistance to TMZ in GBM. **a** Clonogenic assay in four independent U87 sh-DDX39B-expressing clones or a non-targeting control treated with vehicle or TMZ at indicated concentrations. Data show mean value from triplicate samples relative to untreated cells, ± SD, repeated. **b** Trypan blue assay in GBM44 GCSs stably expressing sh-DDX39B or non-targeting control treated with TMZ (100 μM, 72 h). Data show percentage of dead cells normalized to vector control from triplicate samples, ± SD, repeated. Inset: IB with anti-DDX39B. **c** Clonogenic assay in U87 cells stably expressing EV or DDX39B treated with vehicle or TMZ at the indicated concentration. Data show mean value relative to untreated cells, ± SD, repeated. Inset: IB with anti-DDX39B. **d** Trypan blue assay in GBM34 GSCs stably expressing EV or DDX39B treated with vehicle or TMZ (100 μM, 72 h). Data show percent dead cells from triplicate samples ± SD, normalized to EV-expressing cells treated with vehicle, repeated with similar results. *P < 0.05, **P < 0.01 (two-tailed *t* test)

As NF-κB plays a prominent role in the cytotoxic response to DNA damage, we examined whether modulating DDX39B level altered the response to the alkylating agent, temozolomide (TMZ). *DDX39B* was knocked down in U87 GBM cells, and clonal survival examined in multiple independent sh-DDX39B clones. Loss of *DDX39B* consistently resulted in significantly greater survival following treatment with TMZ compared to control (Fig. 4a). We next examined induction of cell death in patient-derived GSCs. In GBM44 GSCs, TMZ treatment induced significantly less cytotoxicity following knockdown of *DDX39B* than that seen with control shRNA (Fig. 4b). Consistent with the ability of DDX39B loss to attenuate cytotoxicity, overexpression of DDX39B in U87 GBM cells resulted in decreased clonal survival following TMZ treatment compared to control (Fig. 4c). Moreover, in GBM34 GSCs that have low basal DDX39B and were very resistant to TMZ, overexpression of DDX39B enabled induction of cytotoxicity by TMZ (Fig. 4d). These results indicate that DDX39B promotes cytotoxicity in response to alkylating chemotherapy.

### DDX39B is regulated by sumoylation and PIASx-β

**Fig. 5:**
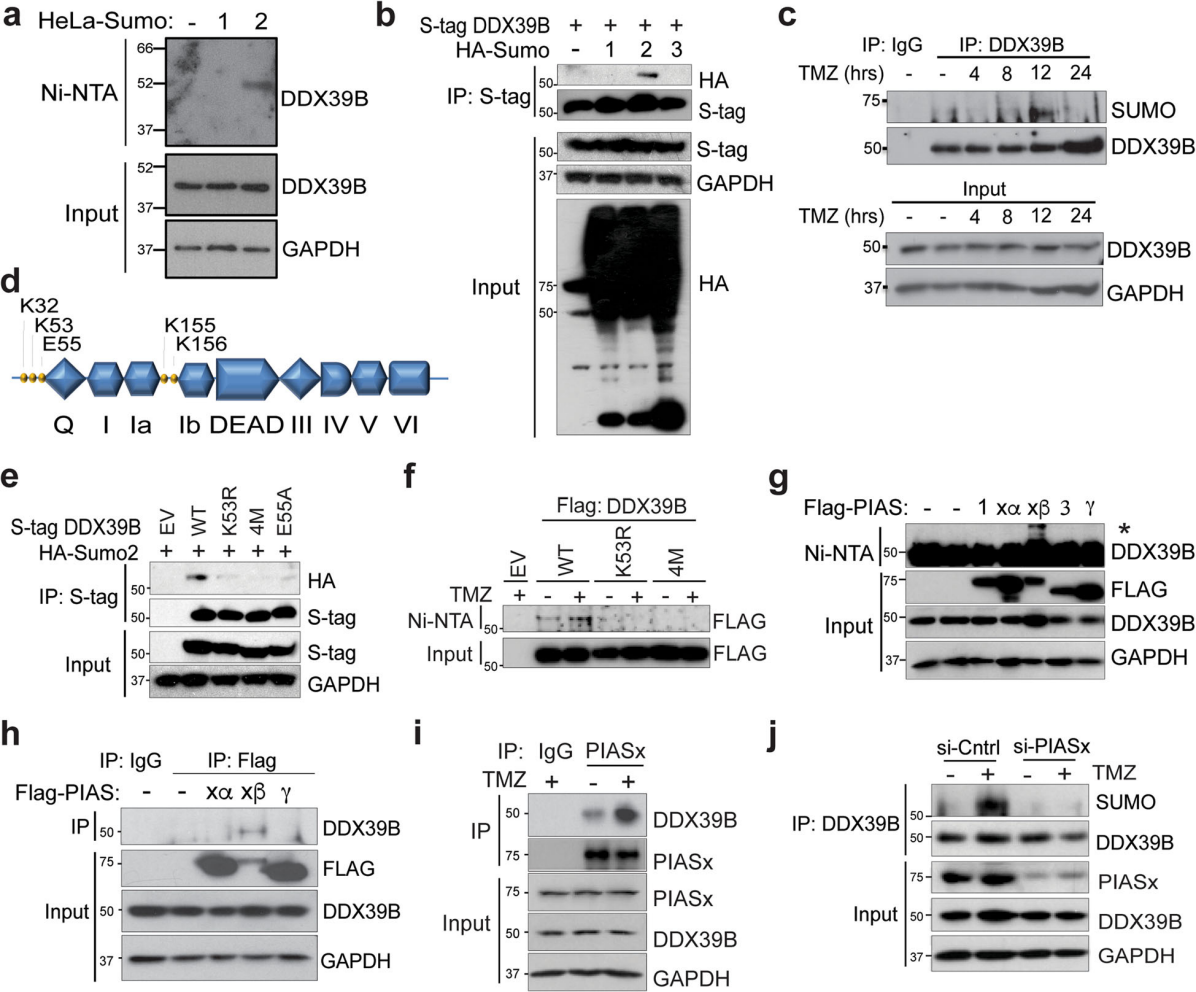
DDX39B is modified by SUMO2 following TMZ treatment. **a** Nickel column (Ni-NTA) pull-down in HeLa cells expressing empty vector, 6His-Sumo1 or 6His-Sumo2. Immunoblot (IB) with anti-DDX39B. Input lysate also probed with anti-DDX39B or anti-GAPDH control. **b** Co-immunoprecipitation (Co-IP) in 293T cells transfected with S-tag DDX39B and HA-Sumo1, HA-Sumo2, or HA-Sumo3. IP performed with S-agarose and IB with anti-HA. Input lysate was probed as shown. **c** Co-IP in U87 cells treated with TMZ (100 μM) for the indicated time. IP with anti-DDX39B or IgG control and IB with anti-SUMO and anti-DDX39B. Input (lower blot) was probed as shown. **d** Schematic of predicted Sumo sites in DDX39B. DEAD-box domains indicated. **e** Co-IP in 293T cells transfected with empty vector (EV) or S-tag DDX39B (wild-type or the indicated mutant) and HA-Sumo2. IP performed with S-agarose followed by IB with anti-HA. **f** Ni-NTA pull-down in HeLa-SUMO2 cells transfected with EV or FLAG-DDX39B (wild-type or mutant) as indicated and treated with vehicle or TMZ (100 μM, 12 h). IB with anti-FLAG. **g** Ni-NTA pull-down in HeLa-SUMO2 cells transfected with empty vector or the indicated FLAG-PIAS protein. IB performed with anti-DDX39B. Input lysate was probed with the indicated antibody. Asterisk, sumoylated band. **h** Co-IP in U87 cells transfected with empty vector or Flag-PIAS proteins as indicated. IP with anti-FLAG and IB with anti-DDX39B. Input was probed with the indicated antibody. **i** Co-IP in U87 cells treated with vehicle or TMZ (100 μM, 12 h). IP performed with anti-PIASx or IgG control and IB with anti-DDX39B antibody. **j** IB in U87 cells transfected with si-PIASx or si-control and treated with vehicle or TMZ (100 μM, 12 h). IP performed with anti-DDX39B and IB with anti-SUMO

The propensity of DDX39B to block NF-κB activity raised the question of how this action is regulated. Sumoylation is a post-translational modification (PTM) that plays an important role in modulating the activity and metabolism of DExD-box helicases [35, 36]. To examine DDX39B sumoylation, we obtained HeLa cells stably expressing His-tagged SUMO 1 and 2 [37]. Using these cells, we saw a unique band in SUMO 2 expressing cells after nickel affinity purification and anti-DDX39B immunoblot (Fig. 5a). This finding was also noted when Flag-DDX39B was overexpressed in these cells (Additional file 1: Fig. S4a). To further examine this, we expressed SUMO 1, 2, and 3 in HEK293T cells. Despite the close homology between SUMO 2 and 3, DDX39B was only modified by SUMO 2 (Fig. 5b). In addition, given the role of DDX39B in the cytotoxic effect of alkylating DNA damage, we examined whether TMZ treatment affected its sumoylation. DDX39B was sumoylated in response to TMZ, and this effect was maximal 12 h after treatment (Fig. 5c).

To identify the sumoylation site, SUMOsp 2.0 software was used [38]. One lysine, K53, was identified within a consensus sumoylation motif (ΨKXE; where Ψ represents a bulky aliphatic residue), and three other lysines, K32, K155, and K156, were found within non-consensus sites (Fig. 5d). We constructed a K53R mutant obliterating the consensus sumoylation site and also an E55A mutant that removed the K53 sumoylation motif without disturbing the lysine itself. We also mutated all four potential sumoylation sites together, 4-mutant (4M). Expression of K53R with HA-SUMO 2 resulted in substantially reduced basal sumoylation of DDX39B (Fig. 5e). Similarly, sumoylation of E55A and 4M was reduced compared to wt. To validate the role of these residues, we used HeLa SUMO 2 cells. Again, mutation of K53 or its motif (E55A) blocked DDX39B sumoylation compared to wt (Additional file 1: Fig. S4b). Although a low level of sumoylation was seen with the mutants in some experiments, there was no difference in sumoylation of K53R and 4M suggesting that K53 was the important site. In addition, mutation of K53 blocked the increase in sumoylation induced by TMZ treatment (Fig. 5f).

To identify the potential E3 ligase involved in DDX39B sumoylation, we noted that in a previous yeast two-hybrid screen, protein inhibitor of activated statx-Beta (PIASx-β) was identified as a factor that interacts with DDX39B/Bat1 [39]. To screen for the potential role of PIAS proteins in DDX39B sumoylation, we expressed five PIAS constructs in HeLa SUMO 2 cells. Only PIASx-β increased the sumoylation of DDX39B (Fig. 5g). Moreover, when overexpressed, only PIASx-β not PIASx-α or PIASγ interacted with DDX39B (Fig. 5h). We also examined endogenous PIASx using a general PIASx antibody and found that this ligase interacted with DDX39B (Fig. 5i), and consistent with its ability to induce DDX39B sumoylation, treatment with TMZ increased the interaction of PIASx with DDX39B (Fig. 5i). In addition, knockdown of *PIASX* expression blocked both basal and TMZ-induced sumoylation of DDX39B (Fig. 5j). Together, these results indicate that DDX39B is sumoylated by a mechanism involving PIASx-β and that sumoylation occurs at K53.

### DDX39B sumoylation promotes its degradation

**Fig. 6:**
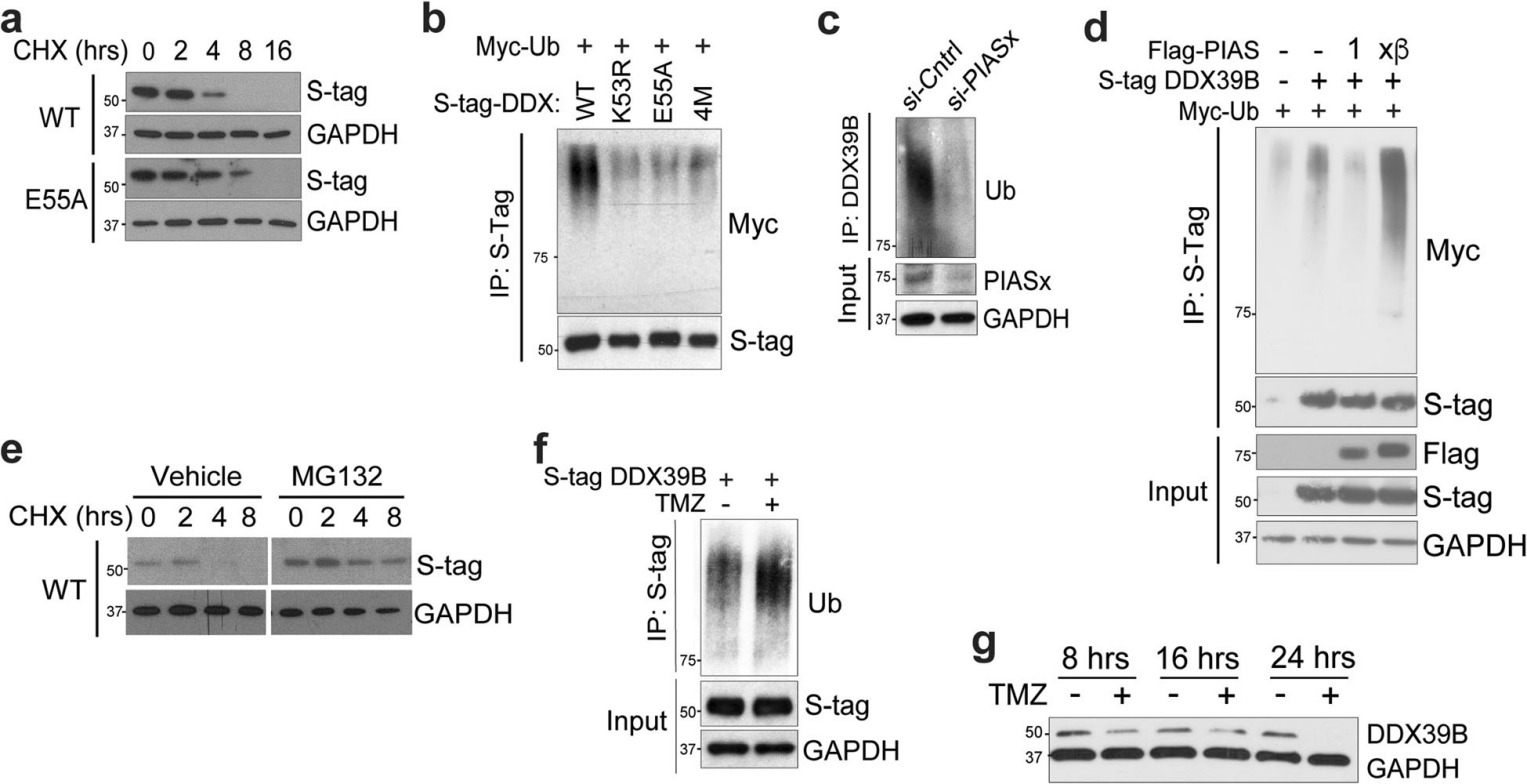
DDX39B stability is regulated by SUMO-dependent ubiquitination. **a** IB in U87 cells stably expressing S-tagged wild-type DDX39B (WT) or E55A-DDX39B (E55A) in the presence of cycloheximide (CHX). Cells were harvested at the indicated time after CHX treatment and IB performed with anti-S-tag or anti-GAPDH antibody. **b** IB in 293T cells transfected with S-tagged DDX39B (WT or the indicated mutant) and Myc-ubiquitin (Myc-Ub). IP performed with S-agarose and IB with anti-Myc or anti-S-tag. **c** IB in U87 cells transfected with si-PIASx or si-control. IP was performed with anti-DDX39B and IB with anti-ubiquitin. Input was probed with the indicated antibodies. **d** IB in 293T cells transfected with empty vector or S-tag DDX39B, Myc-Ub, and Flag-PIAS1 or FLAG-PIASx-β. IP with S-agarose was followed by IB with anti-Myc. Input was probed with the indicated antibodies. **e** IB in U87 cells expressing S-tagged wild-type DDX39B (WT) in the presence of cycloheximide (CHX) and either vehicle or MG132 (10 μM). Cells were harvested at the indicated time after start of CHX treatment, and IB performed with anti-S-tag or anti-GAPDH antibody. **f** IB in 293T cells transfected with S-tag DDX39B and treated with vehicle or TMZ (100 μM, 12 h). IP was performed with S-agarose and IB with anti-ubiquitin. Input was probed with the indicated antibodies. **g** IB in U87 cells treated with vehicle or TMZ (100 μM) for the indicated time in the presence of CHX with anti-DDX39B

Sumoylation of DExD-box helicases has been linked to transcriptional repression. We examined whether DDX39B sumoylation was required for its ability to inhibit NF-κB. While wt-DDX39B inhibited NF-κB activity, mutation of either K53 or E55 did not significantly alter this effect (Additional file 1: Fig. S4c) suggesting that sumoylation of DDX39B does not directly mediate inhibition of NF-κB. Sumoylation also regulates protein stability. To study the role of sumoylation in modulating DDX39B stability, we examined the kinetics of its degradation in the presence of the protein synthesis inhibitor, cycloheximide (CHX). Whereas the amount of wt-DDX39B protein was decreased at 4 h and disappeared within 8 h, mutant DDX39B was not decreased until 8 h (Fig. 6a and Additional file 1: Fig. S4d). Notably, treatment with TMZ did not alter DDX39B mRNA expression (Additional file 1: Fig. S4e). These findings suggested that blocking K53 sumoylation increased the stability of DDX39B protein. As protein stability is associated with poly-ubiquitination, we examined ubiquitination of DDX39B. Consistent with the increased stability of DDX39B sumo-mutants, these mutants had decreased basal poly-ubiquitination compared to wt (Fig. 6b).

We next examined the role of PIASx in this response and found that knockdown of *PIASX* reduced DDX39B poly-ubiquitination compared to control (Fig. 6c). Subsequently, using overexpression studies, we found that while PIAS1 did not increase DDX39B ubiquitination, PIASx-β substantially increased ubiquitin addition (Fig. 6d). Moreover, in the presence of the proteasome inhibitor, MG132, the stability of wt-DDX39B was substantially extended (Fig. 6e). These results indicated that sumoylation of DDX39B at K53 in the presence of PIASx-β resulted in its poly-ubiquitination and proteasomal degradation. Finally, as TMZ also promoted sumoylation of DDX39B, we examined its effect on ubiquitination and found that treatment with TMZ increased DDX39B poly-ubiquitination (Fig. 6f). Consistent with this, TMZ treatment also led to decreased DDX39B protein stability (Fig. 6g). In sum, these findings indicate that DDX39B protein abundance is regulated by sumoylation-dependent ubiquitination and that alkylation damage leads to a decrease in DDX39B protein.

### DDX39B inhibits expression of secreted factors associated with the extracellular matrix, migration, and angiogenesis

**Fig. 7:**
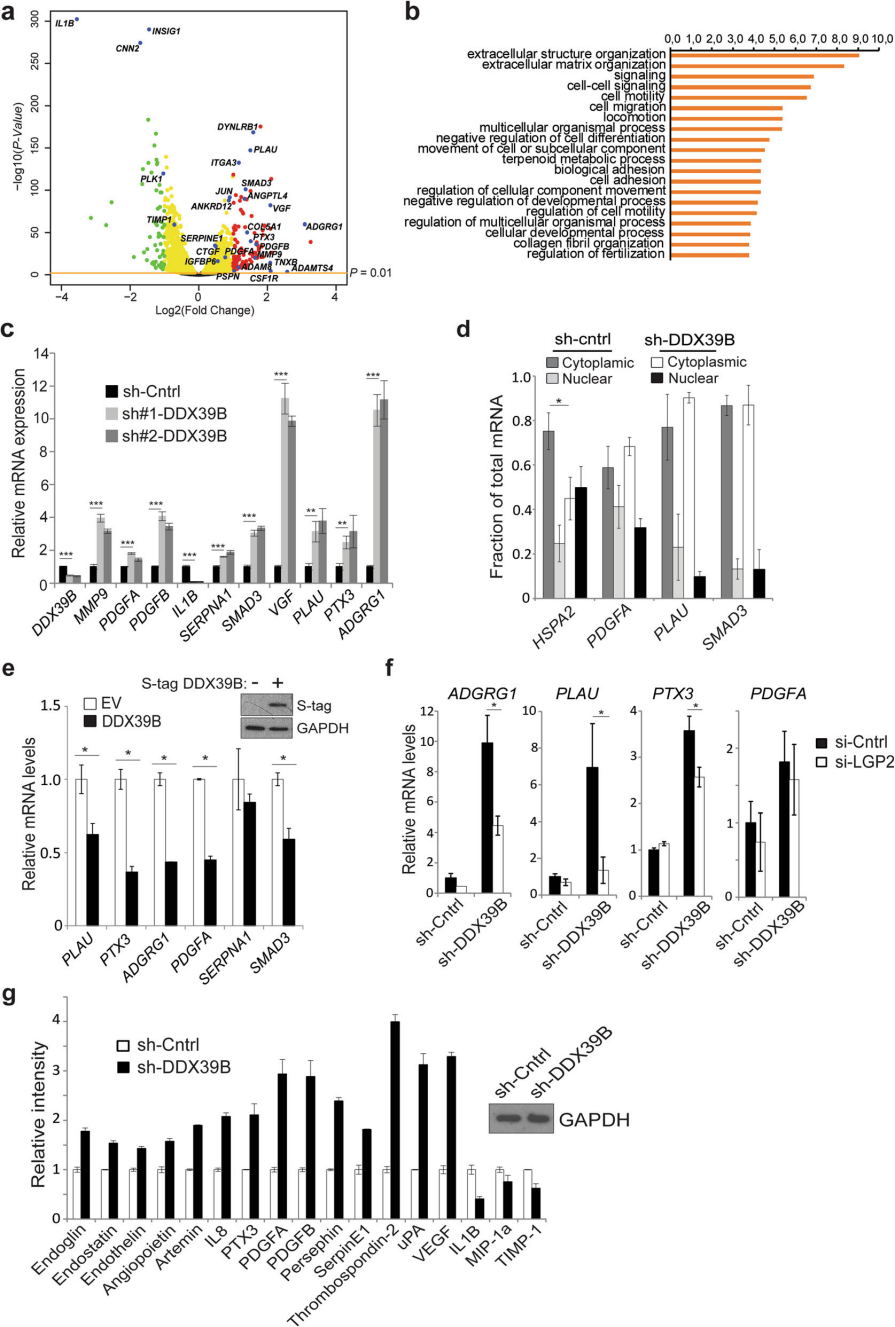
DDX39B inhibits expression of factors associated with the ECM, migration, and angiogenesis. **a** Volcano plot of differentially expressed genes in RNA-seq analysis of GBM44 GSCs expressing two sh-DDX39B constructs compared to a non-targeting control. Red and green dots represent genes up- and downregulated, respectively, in sh-DDX39B cells compared to sh-control. Cutoffs were set at adj. *P* value < 0.01 (orange line) and log2 (fold change) > 1 or < − 1. Blue dots are specific highlighted genes. **b** GO term enrichment among genes upregulated in GBM44 GSCs expressing sh-DDX39B compared to sh-control. *P* values (-log10) of enrichments shown on upper *x*-axis. **c** Quantitative PCR (qPCR) analysis of mRNA expression of indicated genes in GBM44 GSCs. Data show mean value relative to *GAPDH* from three independent experiments, ± SEM, normalized to sh-control. **d** qPCR analysis of mRNA of indicated genes in nuclear and cytoplasmic fractions of cells expressing sh-DDX39B or sh-control. Data show mean value from two independent experiments, ± SEM relative to *RNU1-1*, an abundant small nuclear RNA. **e** qPCR analysis of mRNA in GBM34 GSCs expressing S-tag DDX39B or empty vector (EV). Data show mean value of triplicate samples relative to *GAPDH*, ± SD and normalized to EV-expressing sample. Repeated with similar results. Inset: IB with anti-S-tag. **f** qPCR analysis of indicated genes in GBM44 GSCs expressing sh-DDX39B or a non-targeting control, transfected with si-LGP2 or si-control. Data show mean value of triplicate samples relative to *GAPDH*, ± SD and normalized to sh-control. Repeated with similar results. **g** Quantification of proteins in GBM44 GSCs expressing sh-DDX39B or sh-control using a membrane-based antibody array. Data show fold change of the indicated protein in sh-DDX39B cells compared to sh-control. Inset: IB with anti-GAPDH demonstrates equal protein loading on membranes. *P < 0.05, **P < 0.01, and ***P < 0.001 (two-tailed *t* test)

The interaction of DDX39B with the PRR response raised the question of whether DDX39B modulated innate immune signaling in general. To begin to study this, we examined expression of interferon beta (*IFNB1*), a primary interferon-stimulated gene (ISG). Loss of *DDX39B* in GBM cells did not significantly alter *IFNB1* expression (Additional file 1: Fig. S5a), suggesting that despite inhibition of NF-κB, DDX39B did not modulate general interferon signaling. Given this finding, to more comprehensively study the DDX39B-dependent response, we examined genome-wide expression in GBM. Using GBM44 GSCs that express high levels of DDX39B, we studied differential gene expression following knockdown of *DDX39B* compared to control (Fig. 7a and Additional file 2: Table S1). Gene ontology (GO) term analysis of the most significantly altered genes (FDR < 0.01) revealed that genes associated with the ECM and migration were among the most significantly upregulated (Fig.7b), while transcripts associated with cytokine-mediated signaling were downregulated (Additional file 1: Fig. S5b). In addition, we interrogated the list of differentially expressed genes (DEGs) with known NF-κB target genes. Of the 430 NF-κB target genes identified by the Gilmore lab [40], 117 were present in the DEGs from our analysis (Additional file 2: Table S1) underlining the relevance of DDX39B to regulation of NF-κB. Consistent with the lack of change of *IFNB1*, no general change in innate immune pathways or IGSs was seen. To validate the RNA-seq data, we performed qPCR analysis following *DDX39B* knockdown and confirmed the changes in expression of many of the most significantly altered genes (Fig. 7c). In addition, we found similar changes in the expression of several of the upregulated genes in a distinct GBM cell line (Additional file 1: Fig. S5c).

To further study the role of DDX39B, we noted that in a previous study, genome-wide analysis had been performed in HeLa cells following knockdown of *DDX39B* [24]. In that study, differential gene expression was not reported. We analyzed the raw data from that study (GSE94730). Notably, GO term analysis revealed that similar to our findings, the subgroups of factors most significantly upregulated with loss of *DDX39B* included genes that were either secreted or associated with the ECM (Additional file 1: Fig. S5d and Additional file 3: Table S2). The primary terms linked to downregulated genes included GTP associated factors (Additional file 3: Table S2).

Given that the RNA-seq studies were performed on whole cell lysates and that loss of DDX39B leads to nuclear retention of a subset of mRNAs, we examined whether the upregulated transcripts demonstrated any change in cellular distribution. To this end, mRNA levels were quantified in nuclear and cytosolic fractions. Although loss of *DDX39B* attenuated the cytoplasmic fraction of *HSP70*, an mRNA previously shown to be exported by DDX39B [41], it did not significantly change the amount of cytoplasmic *PDGFRA*, *PLAU*, or *SMAD3* (Fig. 7d). These results indicated that transcripts upregulated with loss of *DDX39B* were also exported out of the nucleus where they could be translated. To further study this response, we expressed exogenous DDX39B in GBM34 GSCs that have low basal DDX39B. Overexpression of DDX39B decreased the level of several of the genes that were identified in the RNA-seq analysis (Fig. 7e) validating the ability of DDX39B to attenuate expression of these factors. In addition, to determine whether the link between DDX39B and LGP2 was important in modulating endogenous gene expression, we examined expression of several genes following knockdown of *LGP2* in the presence of sh-DDX39B. Notably, depletion of *LGP2* reduced the increase in *ADGRG1*, *PLAU*, and *PTX3*, but not *PDGFA*, induced by *DDX39B* loss (Fig. 7f). These results indicate that DDX39B attenuates the expression of certain genes involved in the ECM and angiogenesis in association with LGP2.

Finally, as DDX39B regulates multiple aspects of mRNA metabolism including splicing, we were interested in whether the changes in mRNA expression were reflected by similar alterations at the protein level. To screen for changes in multiple proteins, we used a membrane-based array that contained antibodies against many of the factors that were significantly altered in the RNA-seq analysis. Knockdown of *DDX39B* resulted in an increase in the protein abundance of many of the factors that were increased at the mRNA level (Fig. 7g and Additional file 1: Fig. S5e). Moreover, the transcript that was most significantly downregulated on RNA-seq following loss of *DDX39B*, *IL1B*, was also strongly decreased at the protein level (Fig. 7g). These findings confirmed that the mRNA changes seen with loss of *DDX39B* were recapitulated at the protein level. Notably, the proteins induced with loss of *DDX39B* act to promote angiogenesis, cell migration, and interaction with the ECM (e.g., VEGF, uPA, IL6, PTX3, and PDGFB), supporting the mRNA data and indicating that in GBM cells, DDX39B attenuates expression of secreted factors that are associated with these processes.

## Discussion

In this report, the DExD-box RNA helicase DDX39B was identified as a factor that inhibits NF-κB activity and facilitates cytotoxicity by alkylating chemotherapy. We initially isolated DDX39B because of its propensity to differentially bind κB DNA probes and subsequently found that it inhibited NF-κB in association with the PRR response. Although DDX39B has previously been reported to bind DNA [42], we found that this occurred as a function of the primary nucleotide sequence. Loss of *DDX39B* resulted in increased p65 and IκBα phosphorylation. The increased phosphorylation in the absence of external cytokine stimulation suggested an intrinsic mechanism of activation such as seen with oncogenes or innate immune signaling. To elucidate this pathway, we examined primary MEFs from mice deleted of various components of the innate immune system and found that induction of NF-κB following depletion of *DDX39B* required the PRR response involving LGP2, MAVS, and TRIF. Although these three factors are functionally interrelated in that LGP2 regulates MAVS signaling [43], and MAVS and TRIF together modulate the downstream response [9, 44], DDX39B specifically interacted with LGP2, not MAVS or TRIF. LGP2 is a unique RLR that lacks CARD domains and can both inhibit and activate the response to cytoplasmic dsRNA. While the positive RLR effects of LGP2 were shown to require its ATPase and helicase function [45], its inhibitory effects did not require enzymatic activity or RNA binding [46]. LGP2 was recently reported to inhibit NF-κB activity independent of RNA binding via its interaction with TNF receptor-associated factor (TRAF) proteins [43]. The ability of LGP2 to regulate NF-κB independent of RNA binding is consistent with our finding that loss of DDX39B activated NF-κB without altering cytoplasmic dsRNA content. The lack of increased dsRNA suggested that simple protection of cytoplasmic mRNA is not the mechanism by which DDX39B attenuates NF-κB, a finding supported by the observation that neither Rig-I nor Mda5 were involved in this response. Rather, the data suggested that it was specifically the interaction of DDX39B with LGP2 that was important in mediating the inhibitory effect. In support of this, we found that although DDX39B is primarily a nuclear protein, it is also present in the cytoplasm, the cellular compartment where LGP2 is located.

The increase in NF-κB activity with loss of DDX39B indicated that this pathway might regulate resistance to DNA damaging therapy. Using both established GBM cells and patient-derived GSCs, we found that depletion of DDX39B increased resistance to the alkylating agent, TMZ. While the general increase in NF-κB activity and p65 phosphorylation represents the potential mechanism for this resistance, the interaction of DDX39B with LGP2 may also be relevant to therapy resistance. In this regard, LGP2 was previously identified as a top ranked gene that conferred resistance to DNA damaging therapy in GBM [47]. The finding that LGP2 was required for the increase in NF-κB activity induced by *DDX39B* loss demonstrates a mechanism by which these RNA helicases may interact to regulate response to therapy and indicate that modulating DDX39B or its interaction with LGP2 represents a strategy to improve the response to cancer chemotherapy.

Sumoylation is a common mechanism for regulating RBP action [36]. We found that DDX39B was modified by SUMO 2 and that this PTM occurred at K53. Mutation of K53, or its surrounding motif, substantially blocked sumoylation of DDX39B. Although a faint SUMO signal was seen even when 4 potential sumoylation sites were mutated, mutation of K53 completely blocked sumoylation induced by alkylation damage. Whether the faint SUMO signal represented the promiscuity of sumoylation seen with lysine mutation [48] or actual sumoylation at a different site is unclear. Sumoylation of DDX39B was induced by the SUMO E3 ligase, PIASx-β. Notably, PIASx-β was one of only ten proteins that interacted with DDX39B in an independent yeast two-hybrid screen [39]. K53 sumoylation was not required for inhibition of NF-κB activity; rather, sumoylation promoted DDX39B poly-ubiquitination and proteasome-dependent degradation. Given that DDX39B acts to inhibit NF-κB activity, from a functional perspective, sumoylation-dependent degradation represents a mechanism whereby the inhibitory effect of DDX39B can be regulated to prevent the unopposed inhibition of NF-κB. Interestingly, treatment with TMZ increased DDX39B sumoylation and ubiquitination leading to its degradation. Such a decrease in DDX39B level would lead to treatment resistance, a finding consistent with prior reports that in GBM, DNA damaging therapy results in increased treatment resistance [26, 49].

Despite modulating NF-κB activity via the PRR pathway, loss of DDX39B did not generally induce innate immune signaling. Consistent with this, genome-wide analysis following knockdown of *DDX39B* did not demonstrate an increase in pathways linked to ISGs, a finding that we validated using data from an independent study [24]. We found that the transcripts most significantly upregulated with loss of *DDX39B* included factors that interact with the ECM or promote cellular migration and angiogenesis. Some of these factors required LGP2 for their increase indicating that the interaction of DDX39B with LGP2 was not simply relevant to experimental NF-κB activity, but was also important in the regulation of endogenous genes. Importantly, we found that the change in many of the most significantly altered transcripts was recapitulated at the protein level. Together, these results support the role of DDX39B in attenuating inflammatory signaling and specifically the expression of secreted factors associated with the ECM and angiogenesis.

## Conclusions

Although DDX39B is best known for its role in regulating mRNA splicing and nuclear export, independent studies identified this helicase as Bat1, a factor associated with inflammatory diseases [18–21]. While these disparate actions have not been previously linked, we found that DDX39B acts via the response to dsRNA to inhibit NF-κB activity and reduce expression of factors that regulate the ECM and angiogenesis. This observation, when considered with the role of DDX39B in regulating mRNA processing, supports a model whereby DDX39B binds mRNA in the nucleus, either as part of the splicing or nuclear export machinery, then translocates to the cytoplasm where it interacts with PRRs to attenuate a subset of the inflammatory response (Additional file 1: Fig. S5f). By acting in this manner, DDX39B reduces inflammation induced by endogenously produced RNA. While the increase in inflammation induced by loss of DDX39B likely contributes to autoimmune disease [23, 24], given the importance of NF-κB-mediated inflammation to therapy resistance [50], the increased inflammation seen with decreased DDX39B likely also underlies the resistance to chemotherapy.

## Materials and methods

### Cell lines and reagents

U87, A172, and HEK293T (293T) cells were purchased from American Type Culture Collection. HeLa cells stably expressing empty vector (EV), 6His-SUMO 1 and 6His-SUMO 2, were obtained from Dr. RT Hay and have been described previously [37]. Cells were cultured in DMEM supplemented with 10% FBS and 1% penicillin-streptomycin (ThermoFisher Scientific). GBM34 and GBM44 GSCs obtained from Dr. Mariano Viapiano (Brigham and Women’s Hospital, Boston, MA) were maintained as neurospheres as described [27]. All cell lines were screened for mycoplasma using the ATCC Universal Mycoplasma Detection Kit (catalogue # 30- 121 1012K) every 4 months. Early passage primary MEFs from wild-type, *MyD88*^−/−^, *Rig-I*^−/−^, *Mda5*^−/−^, *Lgp2*^−/−^, *MAVS*^−/−^, *Tmem173*^−/−^, and *Trif*^−/−^ mice were cultured as previously described [51, 52]. All cell lines were authenticated by routine morphological and growth analysis and by western blotting. TMZ was obtained from Sigma-Aldrich. DNA transfection was performed using TransIT LT1 (Mirus) and siRNA transfection with Oligofectamine (Invitrogen). Cycloheximide (CHX) and MG132 were from Cayman Chemical. The following siRNAs were used: siGENOME non-targeting siRNA#3 (Dharmacon), siGENOME Smartpool si-LGP2 (Dharmacon), si-TLR3 (sc-36685, Santa Cruz), and si-PKR (sc-36263, Santa Cruz).

### Plasmid, lentivirus, and CRISPR clone generation

Flag-tagged DDX39B was obtained by cloning DDX39B cDNA into BamHI/XbaI site of pCMV-Tag2b vector. DDX39B mutants, K53R, E55A, and 4M (K32R/K53R/K155R/K156R), were generated by sequential site-directed mutagenesis using Quick-change II Site directed mutagenesis kit (Agilent) according to the manufacturer’s protocol. S-tagged empty vector was generated by annealing oligonucleotide probes containing S-tag coding sequence and inserting into the HindIII/BamHI sites of pcDNA3.1. S-tag DDX39B constructs were generated by cloning the DNA from pCMV-Tag2b into S-tagged pcDNA3.1 using BamHI/XbaI sites. CMV-10-3xFLAG-LGP2 was a gift from Curt Horvath (Addgene plasmid #58681). DDX39B lentiviral expression constructs were generated by cloning DNA from S-tag pcDNA3.1 by HindIII/XbaI restriction sites into EcoRI/XbaI sites of the pLVX-Puro vector (Clontech). For DDX39B shRNA constructs, sequences were obtained from the Broad Institute Database (see Additional file 1: Fig. S1e). Oligonucleotides were annealed and inserted into pLKO.1 using AgeI and EcoRI sites. A scrambled shRNA (sh-control) pLKO.1 vector was obtained from Addgene. Lentivirus production and infection were performed essentially as previously described [27]. Target cells were infected with virus twice using polybrene reagent (EMD Milipore) by spinoculation and selected with puromycin (Sigma) for 2 days at 1.5 μg/ml for MEFs and GBM cells and at 5 μg/ml for patient-derived GSCs.

For DDX39B CRISPR constructs, the sequences of sgRNAs were obtained from the human sgRNA library, Gecko2.0 (DDX39B#1: GATGTGTCACACTCGGGAGT, DDX39B#2: TGTTCTTGTAGACATGCGTC, and non-targeting sgRNA: ACGGAGGCTAAGCGTCGCAA). Oligonucleotides were annealed and inserted into pSpCas9(BB)-2A-Puro (PX459) V2.0 that was a gift from Feng Zhang (Addgene plasmid #62988). For construction of CRISPR clones, U87 cells were transfected with CRISPR vectors and selected with puromycin at (1 μg/ml) for 4 days. Cells were replated on 96-well plates at 1 cell/well to isolate single clones.

### His-DDX39B purification

For purification of HIS-DDX39B, DDX39B cDNA was inserted into pET-45b vector containing a 5′ polyhistidine tag, and protein isolated from BL21 bacteria essentially as previously described [25]. Protein content was quantified and purity confirmed by PAGE and Coomassie staining.

### Luciferase assays

The -1C (IgK) and -1A (NOD2) κB-luciferase reporters have been previously described and contain tandem κB-sites bearing either a C or A in their -1 position [28]. Luciferase assays were performed using the Dual Luciferase Assay Kit (Promega) as previously described [25]. Human cells and MEFs were either infected with sh-DDX39B or sh-control or transfected with EV or DDX39B together with luciferase vectors and *Renilla reniformis* and analyzed at 24 h. For re-expression of LGP2 in *Lgp2*^−/−^ MEFs, cells were first infected with sh-Ddx39b or S-tag-DDX39B and then electroporated, using electroporation buffer (Mirus) and the Amaxa Nucleofector (Amaxa), with Flag-LGP2 or EV together with the -1C reporter and *Renilla*. Data were analyzed at 36 h. All luciferase studies were performed in triplicate and repeated at least twice.

### Clonogenic assay

These were performed as previously described [25] in established GBM cells and stable shRNA and CRISPR clones following treatment with TMZ. Surviving fraction was calculated based on the plating efficiency of untreated cells, performed in triplicate, and repeated at least twice.

### Trypan blue dye exclusion assay

GBM34 and GBM44 GSCs expressing the indicated vector or U87 DDX39B CRISPR cells expressing EV or S-tag DDX39B were plated in equal numbers and treated with TMZ (100 μM for 72 h). Single cell suspensions were prepared in balanced salt solution (DPBS) mixed with 0.4% Trypan blue solution (w/v) at a ratio of 10:1 (v/v). Cell death was calculated as the number of cells that take up dye as a percentage of the total number of cells. Each experiment was performed in triplicate and repeated.

### Immunoblotting and protein array

Immunoblots were performed as previously described [25] with the antibodies indicated. The following antibodies were used: anti-GAPDH (sc-32233, Santa Cruz, 1:10,000 dilution), anti-FLAG (20543-1-AP, Proteintech, 1:10,000 dilution), anti-HA (sc-7392, Santa Cruz, 1:10,000 dilution), anti-S-tag (688102, Biolegend, 1:10,000 dilution), anti-Sumo (PA5-11375, ThermoFisher, 1:1000 dilution), anti-DDX39B (sc-271395, Santa Cruz, 1:1000 dilution or TA501186, Origene, 1:1000 dilution), anti-PIASx (sc-166494, Santa Cruz, 1:1000 dilution), anti-Myc (sc-40, Santa Cruz, 1:10,000 dilution), anti-ubiquitin (sc-9133, Santa Cruz, 1:1000 dilution), anti-phospho-IκBα (sc-8404, Santa Cruz, 1:1000 dilution), anti-IκBα (sc-203, Santa Cruz, 1:1000 dilution), anti-phospho-S536-p65 (93H1, Cell Signaling, 1:1000 dilution), anti-p65 (D14E12, Cell Signaling, 1:1000 dilution), anti-MAVS (14341, Proteintech, 1:1000 dilution), anti-MDA5 (70R-17888, Fitzgerald, 1:1000 dilution), anti-Rig-I (D14G6, Cell Signaling, 1:1000 dilution), anti-LGP2 (sc-373827, Santa Cruz, 1:1000 dilution or 70R-16832, Fitzgerald, 1:1000 dilution), anti-Trif (4596S, Cell Signaling, 1:1000 dilution), and anti-p50 (D4P4D, Cell Signaling, 1:1000 dilution). For co-IP analyses, S-tag DDX39B was pulled down using S-beads (69704-3, Sigma, 30 μl per sample) for 3 h at 4 °C prior to IB. For endogenous Co-IP, IP was performed with anti-DDX39B (H6-X, Santa Cruz) or anti-PIASx (D12, Santa Cruz) antibody overnight at 4 °C. All immunoblots are representative of at least two separate experiments.

For analysis of human angiogenesis protein array, whole cell lysates were isolated from the indicated cells and applied to membranes according to the manufacturer’s protocol (R&D Systems). Equal loading of samples was determined by immunoblot analysis with anti-GAPDH. Image quantification was performed using ImageJ and Protein Array Analyzer for ImageJ, dot by dot, as described in the manual.

### Cell fractionation and electrophoretic mobility shift assay

Cell fractionation was performed as described before [25]. Briefly, cells were resuspended in cell lysis buffer (20 mM Tris-HCl, pH 7.5, 10 mM KCl, 1.5 mM MgCl2, 1% Triton X-100, and protease inhibitor cocktail) on ice for 10 min. Triton-X was added at final concentration of 0.5%, and cells were vortexed for 15 s. The cytoplasmic fraction was collected by centrifuging the lysate at 1000*g* for 3 min and cleared by centrifugation at 15,000 rpm for 10 min. The nuclear pellet was lysed in nuclear lysis buffer containing protease inhibitor cocktail.

Oligonucleotide probes were obtained from IDT (Additional file 4: Table S3) and labeled with [γ-^32^P] ATP using T4 polynucleotide kinase. Bacterially expressed DDX39B was used for electrophoretic mobility shift assay (EMSA). One hundred-fold molar excess of unlabeled NF-κB probe was included in competition assay. Complexes were separated by electrophoresis on non-denaturing 5% acrylamide gel and assayed by autoradiography. Experiments were performed at least twice.

### DNA and nickel column pull-down

DNA pull-down analysis was performed as described [53] using either a -1C or a -1A biotin-labeled κB DNA probe. Nuclear extracts of U87 cells were pre-cleared with non-specific biotin-labeled DNA for 30 min together with streptavidin breads (1420S, New England Biolabs). Samples were then incubated with the biotin-labeled DNA probes for 2 h followed by incubation with streptavidin beads for additional 2 h. Beads were washed three times and following SDS-PAGE analyzed by silver staining using the Silver Stain Kit (Biorad). The differentially bound band was then cut out and sent for mass spectrometry analysis by nano-LC-MS/MS. Nickel column pull-down analyses were performed as previously described [37]. Briefly, His-SUMO 1 and 2 HeLa cells were lysed and imidazole and β-mercaptoethanol added to final concentration of 5 mM and 10 mM, respectively. Ni-sepharose beads (GE Healthcare) were used for pull-down for 3 h at 4 °C. Samples were eluted and analyzed by SDS-PAGE.

### RNA isolation and real-time qRT-PCR

Total mRNA was isolated using TRIzol reagent (Ambion) or with RNA mini-prep kit (Zymo). For RNA fractionation experiments, fractionation was performed as described above with exception that buffers were supplemented with RNAseOut (Invitrogen) and DTT 1 mM. RNA concentration was determined using NanoDrop, and cDNA was generated using iScript (Biorad) or cDNA synthesis kit (Applied Biosystems). Quantitative real-time PCR (qPCR) was performed as described previously using EvaGreen SYBR Green (BullsEye) [27]. For analysis of whole cell mRNA, raw Ct values were first normalized to the expression of *GAPDH* and then to the control samples. For studies of nuclear and cytoplasmic mRNA, values were normalized to the abundant small nuclear RNA *RNU1-1*. Experiments were performed in triplicate and repeated at least twice.

### RNA-seq analysis

GBM44 cells were infected with lentiviral vectors expressing sh-DDX39B or sh-control, and triplicate biological samples cultured for 48 h before RNA isolation. Total RNA was extracted using TRIzol reagent followed by DNase treatment and purification. RNA integrity and quantity were assessed using an Agilent 2100 Bioanalyzer (Agilent Technologies), and total RNA (1 μg) was processed for mRNA enrichment with Magnetic mRNA Isolation Kit (NEB). The isolated mRNA from each sample was used to prepare cDNA libraries using NEBNext Ultra RNA Library Prep Kit for Illumina (NEB), and all steps were completed according to the manufacturer’s manual. The cDNA libraries were single end sequenced (1 × 50 bp) on Illumina HiSeq 4000 platform at the University of Chicago genomics core facility. RNA-seq data were processed to generate pre-processed FATSQ files exhibiting the same indexes, followed by a merge-process using CASAVA Pipeline (Illumina). Data was deposited at the NCBI GEO repository (GSE129542). The FASTQ sequences were then aligned to a reference human genome annotation (GENCODE human v29). The alignment was performed using HISAT2 software (version 2.1.0) with default parameters. The aligned sequences were further sorted with Samtools (version 1.9). Expression quantification counting was performed using HTSeq-count (version 0.11.1) using a reference human genome annotation (GENCODE v29) at default union mode. The count matrix was then normalized, and differentially expressed genes identified with the DESeq2 package (version 1.22.2) in R/Bioconductor software (version 3.8). The GO analysis was performed with Gorilla online tool.

RNA-Seq data (GSE94730) from HeLa cells expressing si-DDX39B were previously published [24]. The raw sequencing read FASTQ files from this study were downloaded from the NIH Sequence Read Archive (SRA) with accession number SRP099127. FASTQ sequences were then aligned to a reference human genome annotation (GENCODE human v29) as described above except that pair-end alignment parameter was used. The downstream gene expression analysis was then performed with the same pipeline using default parameters.

### Immunofluorescence staining

Cells were plated on chamber slides and fixed with paraformaldehyde for 10 min. After blocking with 5% BSA in 0.1% Triton, slides were incubated overnight at 4 °C with the following primary antibodies: rabbit anti-p50 (1:300) (13586S, Cell Signaling), rabbit anti-p65 (1:300) (D14E12, Cell Signaling), or mouse anti-double-stranded RNA (1:50) (MABE1132, Millipore). After washing with PBS, slides were incubated with Alexa Fluor 488 secondary antibody (Life Technologies) for 2 h at room temperature and then rinsed and cover-slipped with mounting medium containing DAPI (Fisher Scientific). For negative controls, primary antibody was replaced with appropriate serum.

Fluorescence images were captured on a Zeiss Axiovert 200M microscope. DAPI and AlexaFluor 488 images were captured using sequential acquisition to give separate image files for each. At least three high power fields (20–40×) were selected by viewing DAPI staining. This approach provided data on at least 100 cells per field. Quantification was calculated from samples plated in triplicate by counting all positive cells in each field per run and averaging their total number in a blinded manner. The percentage of these among the total cells was then reported. Each experiment was repeated at least twice with similar results.

### Statistical analysis

In vitro and other studies where indicated were analyzed by two-tailed Student’s *t* test with significance taken as *P* < 0.05.

## Supplementary information

**Additional file 1:Figures S1- S5**.

**Additional file 2:Table S1.** Analysis of RNA-Seq data from GBM44 GSCs expressing sh-DDX39B or non-targeting sh-control.

**Additional file 3:Table S2.** Analysis of RNA-Seq data from HeLa cells transfected with si-DDX39B or non-targeting control siRNA.

**Additional file 4:Table S3.** Sequences of oligonucleotides used in the study.

## Data Availability

Data generated in this study have been submitted to the NCBI Gene Expression Omnibus (GEO; http://www.ncbi.nlm.nih.gov/geo/) under accession number GSE129542.
