# Supplementary material for: DDX39B interacts with the pattern recognition receptor pathway to inhibit NF-κB and sensitize to alkylating chemotherapy
Source: BMC Biol. 2020 Mar 24;18:32. doi: 10.1186/s12915-020-0764-z (PMC7093963; doi:10.1186/s12915-020-0764-z)
Supplement: Supplementary file 1 — Additional file 1:Figures S1- S5. [file 12915_2020_764_MOESM1_ESM.docx]

**Additional File 1: Figures S1-S5**

**DDX39B interacts with the pattern recognition receptor pathway to inhibit NF-κB and sensitize to alkylating chemotherapy**

Szymon J Szymura, Giovanna M Bernal, Longtao Wu, Zhongqin Zhang, Clayton D Crawley, David J Voce, Paige-Ashley Campbell, Diana E Ranoa, Ralph R Weichselbaum and Bakhtiar Yamini


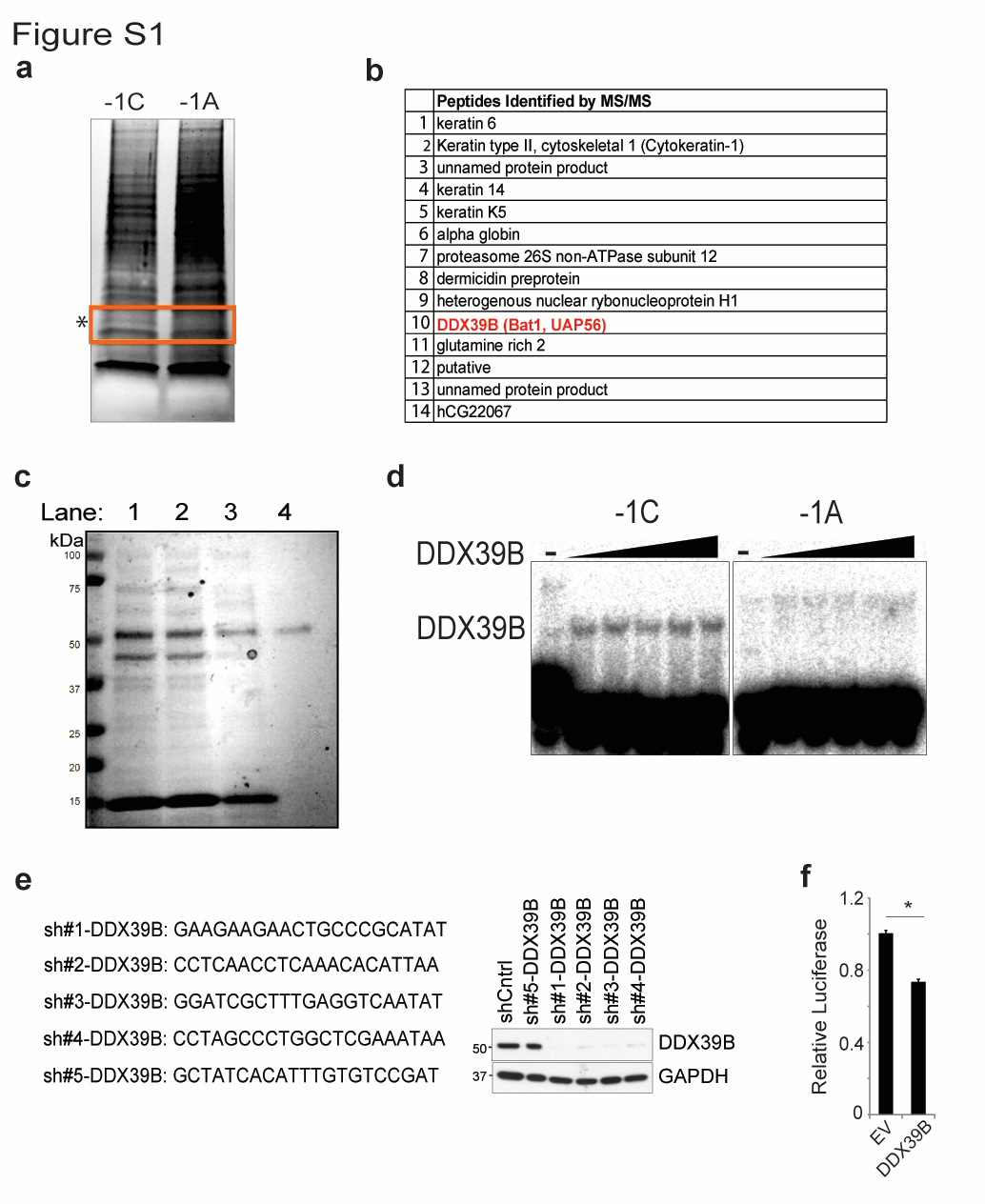


**Figure S1.** (**a**) Silver stain gel. Probes used: -1C: GGGACTTTCC; -1A: GGGAATTTCC. Highlighted band in -1C lane (*) analyzed my MS/MS. (**b**) List of proteins identified by MS/MS analysis. (**c**) Coomassie-stained SDS-PAGE gel of bacterially-expressed His-DDX39B purified on Ni-sepharose column. Samples loaded in each lane: lane 1: expression-induced bacterial lysate; lane 2: Ni-sepharose flow-through after application of lysate; lane 3: column fraction after application of 50 mM imidazole wash buffer; lane 4: eluted fraction after application of 500 mM imidazole. (**d**) EMSA with increasing amounts (0, 15, 30, 90, 150 and 300 ng) of purified DDX39B protein using the -1C or -1A κB probe. (**e**) Sequences of DDX39B shRNA oligonucleotides (left) and representative immunoblot (right) in cells expressing shRNAs probed with anti-DDX39B. anti-GAPDH used as a loading control. (**f**) Luciferase assay in U87 cells with a reporter bearing -1A κB-sites transfected with empty vector or DDX39B. Data show mean value relative to *renilla* normalized to EV, ± SD of triplicate samples. **P* < 0.05.


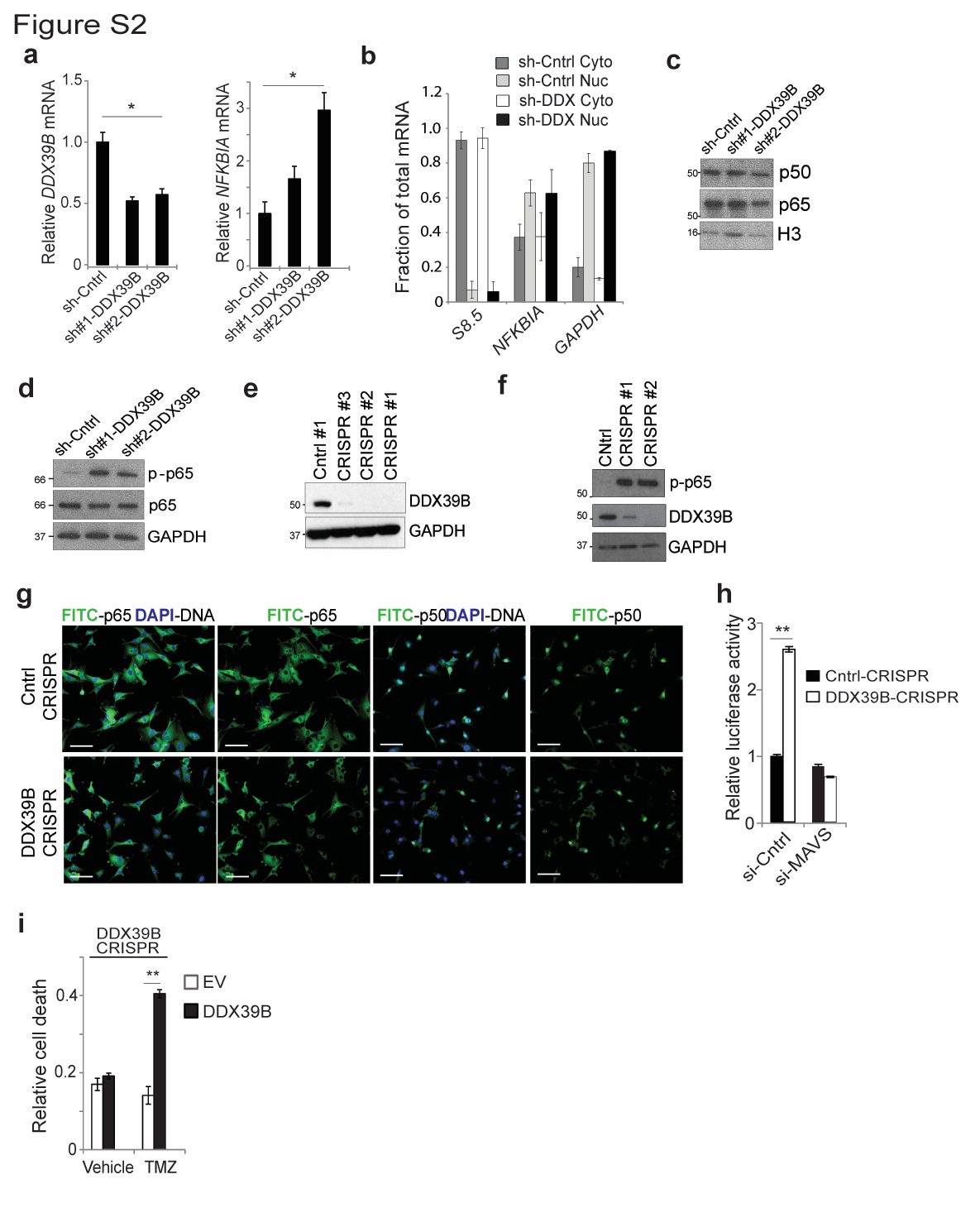


**Figure S2.** (**a**) qPCR analysis of the indicated mRNA in A172 cells expressing sh-DDX39B or non-targeting control. Data show mean value relative to *GAPDH* of two independent experiments, ± SEM normalized to sh-control. (**b**) qPCR analysis of the indicated mRNA in nuclear and cytoplasmic fractions from cells used in B. Data show mean value relative to the small nuclear RNA, *RNU1-1*, ± SEM of two independent experiments. (**c**) IB using nuclear extract from A172 cells expressing sh-DDX39B or control shRNA probed with the indicated antibody. Histone H3 (H3) used as loading control. (**d**) IB with lysate from cells used in B probed with anti-phospho-p65 or anti-p65. (**e**) IB in U87 DDX39B control or CRISPR clones probed with anti-DDX39B. (**f**) IB in U87 DDX39B control or CRISPR clones probed with anti-phospho-p65. (**g**) Immunofluorescence staining with anti-p50 and anti-p65 using U87 DDX39B CRISPR cells. Scale bar, 100 μm. (**h**) Luciferase assay in U87 DDX39B CRISPR or control cells using the -1C κB reporter transfected with si-MAVS or si-control. Data show mean value relative to *Renilla*, ± SD of triplicate samples normalized to control cells. (**i**) Trypan blue assay in U87 DDX39B CRISPR cells expressing either EV or DDX39B treated with vehicle or TMZ (100 μM, 72 hrs). Data show percentage of dead cells from triplicate samples, ± SD. **P* < 0.05, ***P* < 0.01.


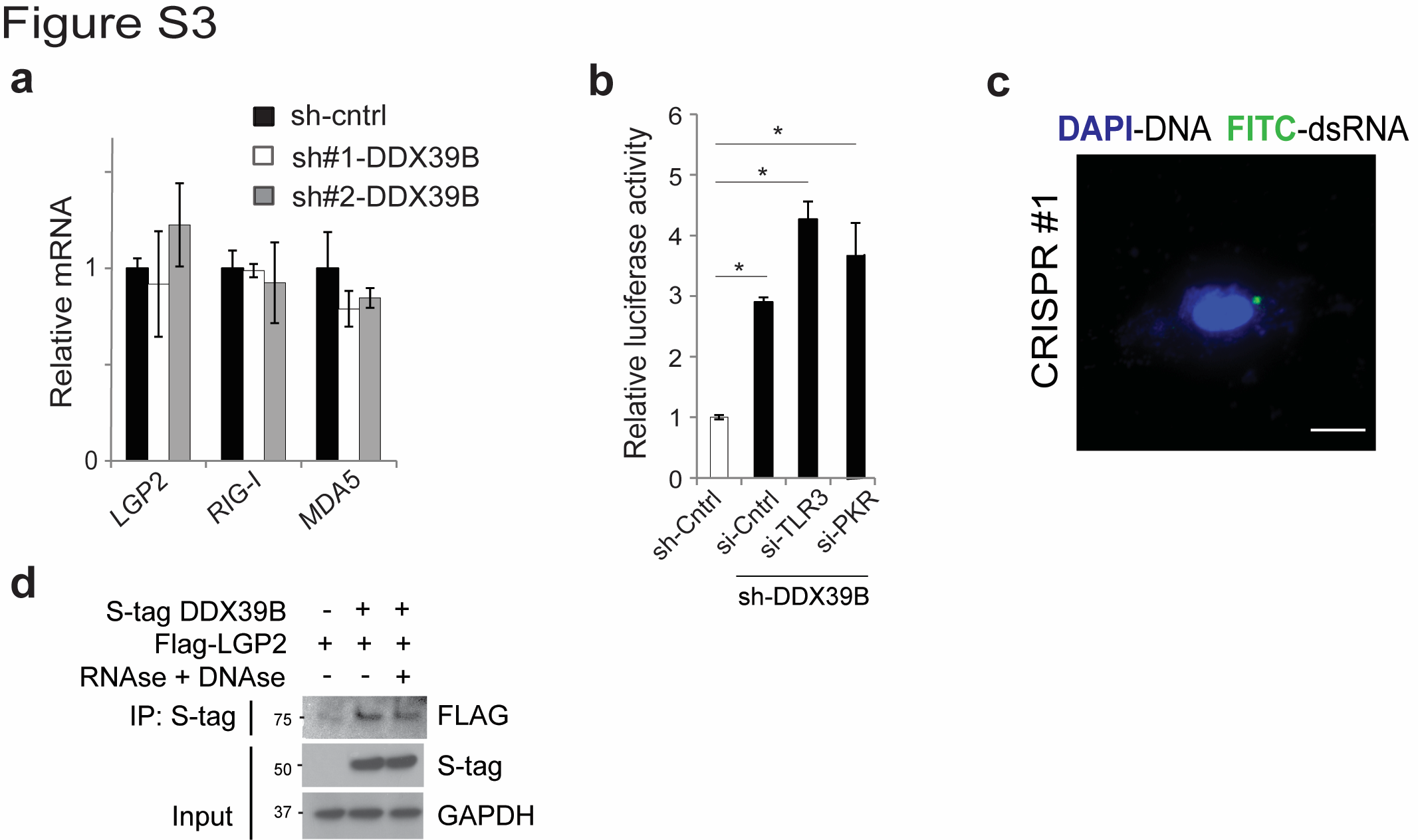


**Figure S3.** (**a**) qPCR analysis of indicated mRNA in A172 cells expressing sh-DDX39B or sh-control. Data show mean value relative to *GAPDH* of two independent experiments, ± SEM normalized to sh-control. (**b**) Luciferase assay using the -1C reporter in U87 cells expressing sh-DDX39B or sh-control. Sh-DDX39B cells were transfected with si-TLR3, si-PKR or si-control. Data show mean value ± SD of triplicate samples normalized to sh-control expressing cells. (**c**) Immunofluorescence staining of dsRNA in U87 DDX39B CRISPR cells associated with Fig 4e. Scale bar, 10 μm. (**d**) Co-IP in 293T cells transfected with empty vector or S-tagged DDX39B and Flag-LGP2. IP with anti-S-tag followed by IB with anti-FLAG. Experiment was performed in the presence and absence of nuclease (RNAse + DNAse). **P* < 0.05.


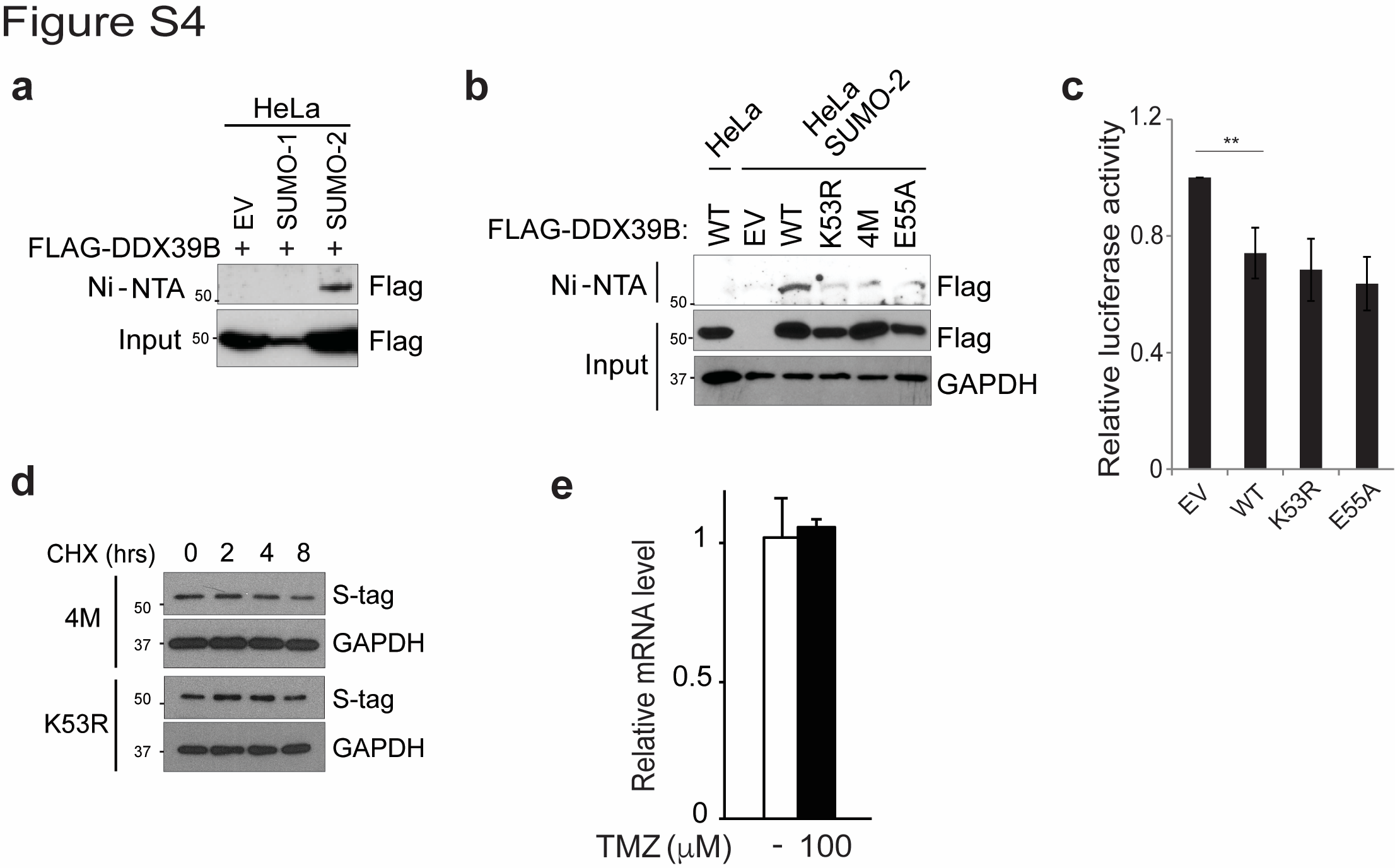


**Figure S4.** (**a**) Nickel column (Ni-NTA) pull-down in HeLa cells stably expressing empty vector (EV), His-SUMO1 or His-SUMO2 transfected with Flag-DDX39B. IB was performed with anti-FLAG antibody. (**b**) Ni-NTA pull-down in HeLa cells expressing wildtype FLAG-DDX39B (WT) or in His-SUMO2 HeLa cells expressing EV, wildtype FLAG-DDX39B (WT) or the indicated FLAG-tagged DDX39B mutant. IB was performed with anti-FLAG and the input probed as shown. (**c**) Luciferase assay using the -1C reporter in U87 cells expressing wt-DDX39B (WT), K53R or E55A mutant DDX39B. Data show mean value, ± SD of triplicate samples, repeated. (**d**) IB in U87 cells stably expressing S-tagged K53R-DDX39B (K53R) or 4M-DDX39B (4M) in the presence of cycloheximide (CHX). Cells were harvested at the indicated time after CHX treatment and IB performed with anti-S-tag or anti-GAPDH antibody. (**e**) qPCR analysis of *DDX39B* mRNA in U87 cells treated with 100 μM TMZ or vehicle (24 hrs). Data show mean value relative to *GAPDH* of two independent experiments, ± SEM normalized to vehicle. ***P* < 0.01.


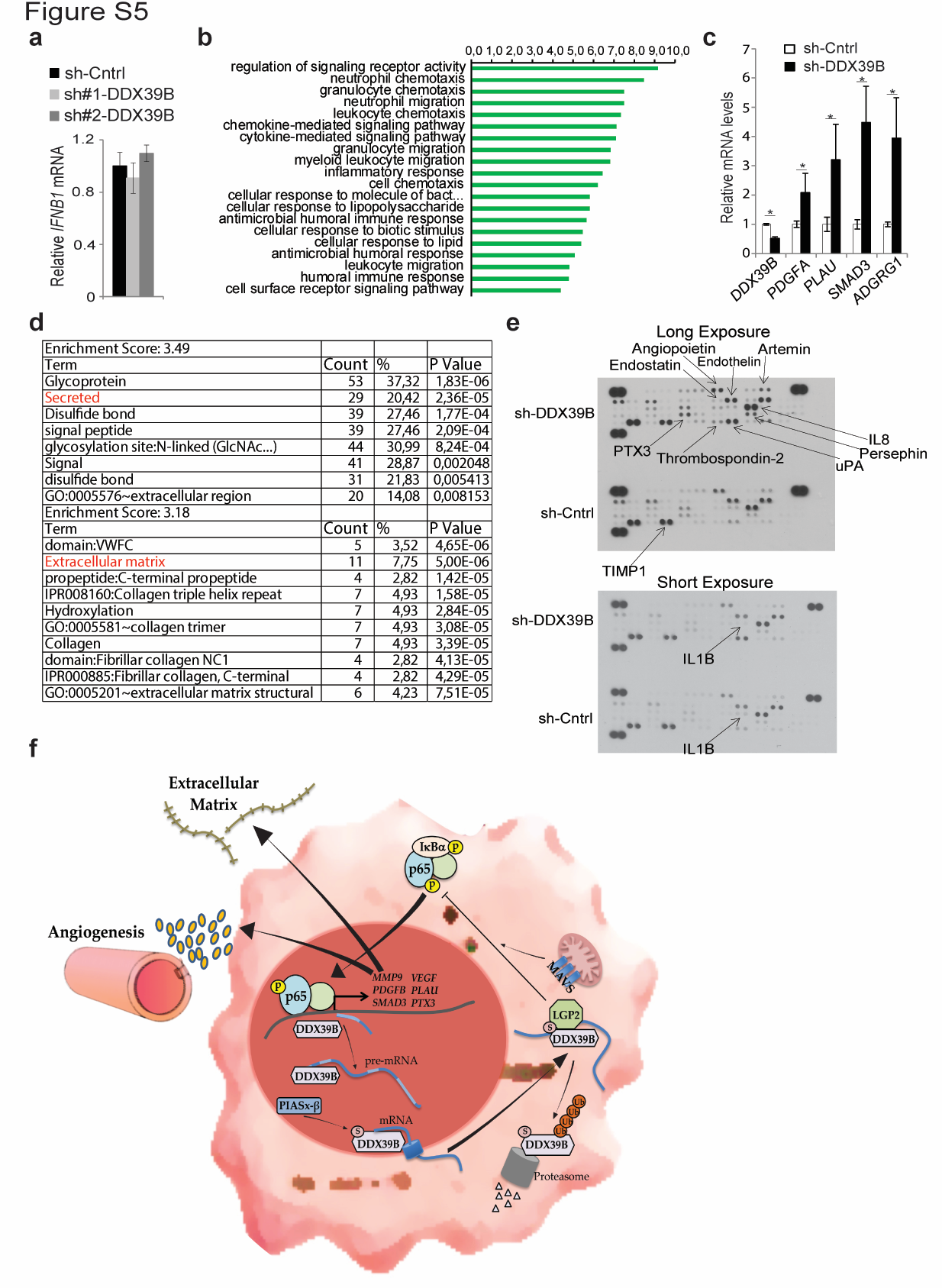


**Figure S5.** (**a**) qPCR analysis of *IFNB1* mRNA in GBM44 GSCs expressing sh-DDX39B or non-targeting control. Data show mean value relative to *GAPDH*, ± SEM of two independent experiments normalized to sh-control. (**b**) GO term enrichment among genes down-regulated in GBM44 GSCs expressing sh-DDX39B compared to sh-control. *P*-values (-log10) of enrichments shown on upper x-axis. (**c**) qPCR analysis of mRNA in A172 cells expressing sh-DDX39B or sh-control. Data show mean value relative to *GAPDH*, ± SEM of two independent experiments normalized to sh-control. (**d**) Top GO term enrichment of up-regulated genes in HeLa cells expressing si-DDX39B compared to si-control from GSE94730. (**e**) Representative membrane from angiogenesis protein array from Fig 7g. (**f**) Schematic model of the role of DDX39B in NF-κB regulation. DDX39B binds pre-mRNA regulating its splicing and subsequent nuclear export. DDX39B interacts with LGP2 leading to inhibition of p65 and IκBα phosphorylation in conjunction with MAVS. This inhibits NF-κB activity and decreases expression of factors that promote angiogenesis, migration and interaction with the extracellular matrix. DDX39B protein is regulated by its sumoylation, in the presence of PIASx-β, and consequent ubiquitin-dependent degradation. **P* < 0.05.
